# Dermal fibroblast cultures recapitulate differences between deermice and mice in their responses to a Toll-like receptor agonist

**DOI:** 10.3389/fimmu.2025.1666789

**Published:** 2025-11-04

**Authors:** Jonathan V. Duong, Aqsa Motiwala, William J. Hotz, Landen Gozashti, Anthony D. Long, Alan G. Barbour

**Affiliations:** ^1^ Department of Microbiology and Molecular Genetics, School of Medicine, University of California, Irvine, Irvine, CA, United States; ^2^ Department of Organismic and Evolutionary Biology, Harvard University, Cambridge, MA, United States; ^3^ Museum of Comparative Zoology, Harvard University, Cambridge, MA, United States; ^4^ Department of Ecology and Evolutionary Biology, School of Biological Sciences, University of California, Irvine, Irvine, CA, United States; ^5^ Department of Medicine, School of Medicine, University of California, Irvine, Irvine, CA, United States

**Keywords:** infection tolerance, innate immunity, cell-autonomous immunity, interleukin-11, secretory leukocyte peptidase inhibitor, endogenous retrovirus, Nrf2, arginine

## Abstract

**Introduction:**

The white-footed deermouse *Peromyscus leucopus* is a primary reservoir for the agents of Lyme disease and other zoonoses in North America and manifests infection tolerance for the bacteria, protozoa, and viruses it hosts. In previous in vivo studies, *P. leucopus* and *Mus musculus* differed in the degree of sickness and profiles of biomarkers after exposure to a bacterial lipopolysaccharide, a TLR4 agonist.

**Methods:**

As an approach for assessing immunity of mammals in nature and for longitudinal studies of colony animals in the laboratory, we evaluated primary dermal fibroblast cultures of *P. leucopus* and *M. musculus* in their short-term responses to a TLR2 agonist lipopeptide using bulk and single-cell RNA-seq.

**Results:**

By single-cell RNA-seq, cultures of both species comprised at least two types of fibroblasts, which were further differentiated in their responses to TLR agonists. With continued passage, the mouse cell population lost viability, while the deermouse cell population spontaneously transformed into a cell line stably maintained under standard conditions. Bulk RNA-seq revealed distinctive profiles for deermouse and mouse cells in arginine metabolism gene expression, high baseline transcription of the antioxidant transcription factor Nfe2l2 (Nrf2) in deermouse fibroblasts, and the transcription of the aging-associated cytokine interleukin-11 in agonist-treated mouse fibroblasts but not deermouse fibroblasts. In the cultures of both species, there was increased transcription of several types of endogenous retrovirus (ERV) and transposable elements (TEs) after exposure to the agonist. The transcribed ERV/TE sequences in *M. musculus* cells were generally longer in length and had greater potential for translation than sequences in treated *P. leucopus* cells.

**Discussion:**

The results indicate the feasibility of this in vitro model for both laboratory- and field-based studies, and that inherent differences between deer mice and mice in cell-autonomous innate immune responses and ERV/TE activation can be demonstrated in dermal fibroblasts as well as the animals themselves.

## Introduction

As Hagai et al. noted, the immune system’s characteristics of both rapid divergence and high cell-to-cell variability “seem to be at odds with strong regulatory constraints imposed on the host immune response: the need to execute a well-coordinated and carefully balanced program to avoid tissue damage and pathological immune conditions” (1). To resolve this apparent paradox, established animal models, principally the house mouse *Mus musculus*, are commonly enlisted. But what if the question could be more profitably addressed using another species (2), such as one that, in its natural environment, thrives while persistently infected with microbes of trans-species transmission potential? Ideally, wild populations of the species would be accessible for field-based studies, and there would be vivarium-bred stock colonies available for laboratory-based studies.

Our nominee as an alternative to the house mouse for this question is the North American species *Peromyscus leucopus*, the white-footed deermouse (3, 4). Mouse-like in appearance and size, this and other deermice, such as *P. maniculatus*, belong to the family Cricetidae, along with hamsters, voles, and woodrats, and not the family Muridae, the taxonomic clade of laboratory mice and rats (5). *Peromyscus leucopus* is abundant in the eastern and central United States and found in a variety of environments. Closed colonies of genetically diverse stock exist at different institutions. As a foundation for forward and reverse genetics, as well as for bulk and single-cell RNA-seq, there are high-quality, chromosome-scale genome assemblies of both *P. leucopus* and *P. maniculatus*, with full annotation and millions of single-nucleotide polymorphisms and other variants that have been catalogued to date (6, 7).

Another rationale for choosing *P. leucopus* is its public health importance as a reservoir for several agents of human zoonoses. Besides the Lyme disease agent, *P. leucopus* is also a natural host for the obligate intracellular bacterium of anaplasmosis, the apicomplexan protozoan of babesiosis, and the flavivirus of Powassan viral encephalitis. Deermice may harbor a given pathogen at loads sufficient for transmission to a vector, but they are largely free of disability or discernible effects on fitness [reviewed in (8)]. Infected with the spirochete *Borreliella burgdorferi*, the principal agent of Lyme disease in North America, *P. leucopus* displays a few of the pathologic changes or inflammation observed in infected *M. musculus.*

This phenomenon is known as infection tolerance (9, 10), which is characterized by immunological and physiological adaptations that minimize the harm from a pathogen’s presence without necessarily reducing its burden. Infection tolerance by this definition subtly differs from “disease tolerance,” which can be thought of as the ability of a host to survive or maintain fitness despite experiencing disease symptoms or pathology (11–13). Disease tolerance is a concept that places more emphasis on damage mitigation and repair mechanisms. Whether we interpret a finding as tolerance of infection or of disease, the phenomenon may have relevance for studies of aging. *Peromyscus leucopus* has a maximum longevity that is two to three times longer than that of *M. musculus* (14, 15). Infection tolerance and greater longevity for their body sizes are characteristics that *P. leucopus* has in common with some bats (16, 17).

These distinctions between deermice and mice inspired our comparative studies of these representatives of two rodent genera. The experimental study design was to induce a host response to a microbial product (18), specifically bacterial lipopolysaccharide (LPS), which is a Toll-like receptor 4 (TLR4) agonist and known to elicit acute systemic inflammation (19, 20). Thus, treated *P. leucopus* had profiles of genome-wide RNA-seq of the blood, spleen, and liver that distinguished it from treated *M. musculu*s. In general, the LPS-treated deermice displayed alternatively activated macrophage polarization rather than the expected classically activated mitigation of the effects of neutrophil activation and restrained type 1 and type 2 interferon responses. More specifically, *P. leucopus* was distinguished from *M. musculus* by an inverted ratio of nitric oxide synthase 2 (Nos2) to arginase 1 (Arg1) transcription, remarkably high transcription of the Slpi gene for secretory leukocyte peptidase inhibitor (Slpi), and relatively diminished interferon expression.

For the present study of *P. leucopus* and *M. musculus*, we aimed to reduce the subject of the experiment to one that retains the characteristics of live animals, yet would also serve for studies of wild animals that could be captured and then released (21). Blood sampling from trapped and then released animals is an established procedure for the study of the immunology or physiology of animals in nature (18). However, assays of whole blood or serum are limited to what a single volume of blood can provide and nothing more. If the spleen is the tissue (22), the capture is a terminal event for the animal. For field and laboratory work on Lyme disease, a specimen commonly obtained has been punch biopsies of ear skin tissue, which are then subjected to culture or quantitative PCR for *B. burgdorferi* bacteria (23, 24).

It occurred to us that this biopsied tissue could also be propagated *ex vivo* in the laboratory, thus allowing for a much expanded set of experiments with further rounds of multiplication. A critique would hold that a culture of cells of a tissue is not evaluable as to its “fitness,” as one could for the organisms themselves in an ecology or evolutionary biology context. From this perspective, a tissue culture cannot manifest infection tolerance per se. However, because infection tolerance is characterizable by specific adaptations (25), some of which are of the measurable sort with isolated tissues or cells, there is justification for proposing primary cultures as *in vitro* correlates of a phenomenon that applies primarily at the organismal or population level.

As a proof-of-principle for *Peromyscus*, we cultivated primary dermal fibroblasts obtained from the ears of heterogeneous stock *P. leucopus* or outbred *M. musculus* in a laboratory setting. Low-passage cultures of skin cells were then exposed to either TLR2 agonist or buffer alone. TLR2, as a heterodimer with TLR1, is the pattern recognition receptor (PRR) for pathogen-associated molecular pattern (PAMP) represented by the bacterial lipoproteins of *B. burgdorferi* (26). The resultant specimens were subjected to bulk and single-cell RNA-seq, for which the reference sets were genome-wide protein-coding sequences of *P. leucopus* and *M. musculus*, as well as full sets of endogenous retrovirus-derived elements of each species (27). We also characterized a spontaneously transformed line of *P. leucopus* dermal fibroblasts that continued in its capacity for proliferation long after an analogous *M. musculus* fibroblast line had ceased to replicate.

## Results

### Primary cultures and serial passages

**Figure 1:**
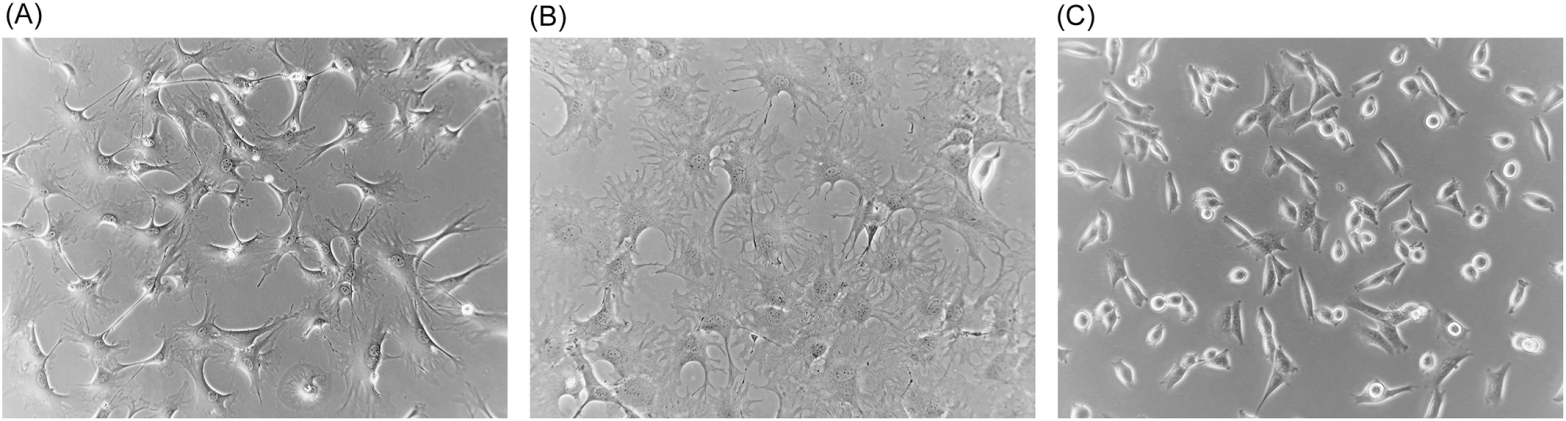
Phase microscopy photographs of cultures of dermal fibroblasts of *Mus musculus* **(A)** and *Peromyscus leucopus* **(B, C)**. **(A, B)** Second passage cells of each species, and **(C)** high passage (P47) transformed cells. Magnification, ×150.

For these experiments, full-thickness samples of freshly excised ears of euthanized *P. leucopus* or *M. musculus* animals were cultivated in a type of medium and under conditions long used for primary fibroblast culture (28). There was no attempt to preserve the spatial characteristics of skin tissue through the addition of growth factors or other supplements. The oxygen concentration was that of an incubator with 5% CO_2_ and at sea level without any adjustments to limit oxidative stress (29). Cells of the original cultures and then of subsequent passages were aliquoted and frozen, thereby preserving the history of the two lineages. Examples of early passage cells from *P. leucopus* and *M. musculus* in culture are shown in **Figure 1**. Successful cultivation of *P. leucopus* dermal fibroblasts was also achieved with single 2-mm punch biopsies of the ears of animals.

In their initial cultures, *M. musculus* cells were confluent on the bottom of the dishes by day 5 to 7, while the *P. leucopus* cells at the same starting inoculum achieved that state only by day 10 to 12. Doubling times for early passage populations of *P. leucopus* cells and *M. musculus* cells were ~56 and ~28 h, respectively. By passage 16 (or ~70 doublings), the time for the *M. musculus* cultures to reach confluency had lengthened to 10–14 days, with a doubling time the same as the *P. leucopus* population at that point. Within a few more passages, the *M. musculus* cells failed to adhere and growth further slowed. The emergence of an established cell line, as can occur with the cultivation of mouse embryo cells (30), was not observed.

Under the same cultivation conditions, by passage 20 (~86 doublings), the time to confluency for *P. leucopus* cells to reach confluency was only 6–8 days, which corresponded to a doubling time of 14 h. Thereafter, the *P. leucopus* cells grew at this higher rate through more than 47 serial passages with no signs of abnormalities in adherence and growth noted for the mouse cells. This apparent adaptation of the *P. leucopus* cells to *in vitro* life was accompanied by a change in cell morphology; they became shorter and more rounded with fewer extensions while retaining their adherence capacity (**Figure 1**). The *P. leucopus* dermal fibroblast culture was considered “spontaneously transformed” thereafter (31).

### Bulk RNA-seq

For the experiments with the lipopeptide TLR agonist Pam3CSK4, the sources of tissue for *in vitro* cultivation were five adult outbred LL stock *P. leucopus* (three females and two males) and five adult outbred CD-1 *M. musculus* (three females and two males) (**Supplementary Table S1**). Second passage (P2) cultures (~16 doublings) at 80%–90% confluency were split into three and, then after growth for 24 h, subjected to the following exposures for 4 h: no treatment control or the lipopeptide TLR2 agonist Pam3CSK4 at 1 or 10 µg/mL. The cDNA libraries were mRNA-stranded and yielded ranges of 1.03–1.42 × 10^8^ paired-end 150 nt (PE150) reads for the 15 P*. leucopus* samples and 1.25–1.63 × 10^8^ reads for *M. musculus*. Reference sets were the protein-coding sequences (CDS) of the genome of a female *P. leucopus* of the LL stock and the reference C57BL/6 genome of a female *M. musculus.* As a consequence of the more comprehensive isoform annotation for the mouse genome to that point, there were four times as many CDS sequences listed for *M. musculus* as for *P. leucopus*. Accordingly, for comparability, the first listed isoform for *M. musculus* was used for the reference set, resulting in non-redundant CDS sets of 22,760 for *M. musculus* and 22,654 for *P. leucopus* (Dryad **Tables D1** and **D2**).

**Figure 2:**
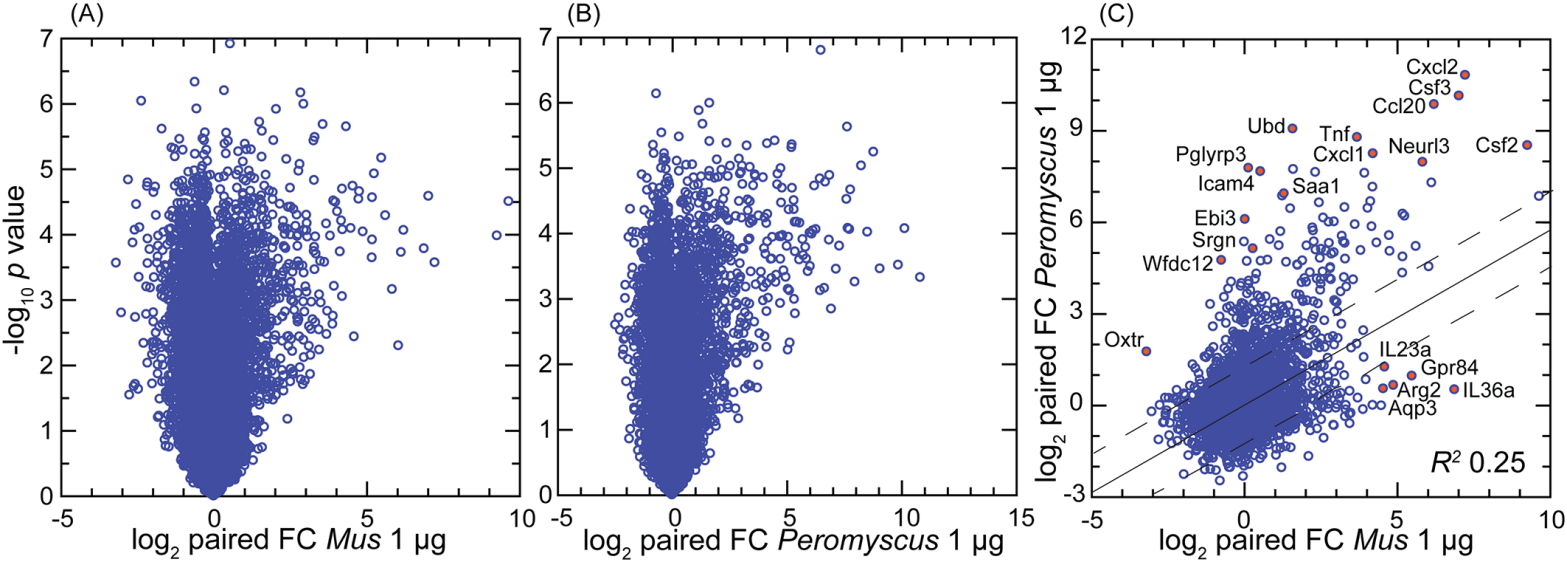
Volcano plot **(A, B)** and scatter plot **(C)** of treatment-to-control fold-change (FC) values from genome-wide bulk RNA-seq of primary dermal fibroblasts of *Mus musculus* **(A)** and *Peromyscus leucopus* **(B)**. The treatment was 1 µg/mL of Pam3CSK4 lipopeptide for 4 h. **(A, B)** Log_10_-transformed paired *t*-test *p*-values are plotted against log_2_-transformed FC for 13,243 *M. musculus* CDS and 12,938 *P. leucopus* CDS with mean TPM ≥10 across all samples. By the criteria of, first, FC ≥2 or ≤0.5 and, second, *p*-value <0.001 (FDR < 0.0025), there were 149 downregulated differentially expressed genes (DEGs) and 261 upregulated DEGs for *M. musculus* **(A)** and 58 downregulated DEGs and 300 upregulated DEGs for *P. leucopus* **(B)**. **(C)** The log_2_-transformed FC values for each species for 14,979 CDS in common are plotted against each other. The linear regression line with 95% confidence interval and coefficient of determination (*R^2^*) is shown. Selected CDS that were upregulated and differentially expressed for each are indicated by name and red fill. Data for analyses are in Dryad **Tables D1** **(B)**, D2 **(A)**, and D3 **(C)**.

Distributions of log-transformed TPM values by cumulative count of genes were similar between species (**Supplementary Figure S1**). There were 14,979 genes in common to both species that met the criterion of TPM ≥10 in at least one of the individual 30 samples (Dryad **Table D3**). There was also similarity between species in the log–log plots of *p*-values and fold changes of paired samples; for both species, the number of DEGs with higher transcription after agonist exposure was greater than the number of DEGs with reduced transcription post-exposure (**Figures 2A, B**). The mean coefficients of determination (*R* ^2^) values in log-transformed reads by CDS between paired values for the 1- and 10-µg per mL concentrations were 0.994 (0.992–0.996) for *M. musculus* and 0.964 (0.927–1.00) for *P. leucopus* (*p* = 0.15), indicating low to negligible dose effect within the 10-fold range of the study (Dryad **Tables D1** and **D2**). Unless otherwise noted, comparisons with paired controls were done with cells exposed to the 1-µg/mL concentration, as likely more representative of the tissue environment in the *B. burgdorferi*-infected skin of *P. leucopus* reservoir hosts.

For the genome-wide RNA-seq for *M. musculus*, it was possible that our choice of the top-listed isoform, i.e., instead of all isoforms for the reference set, and which was done for the sake of comparability with the *P. leucopus* reference set, introduced a design bias. To look for evidence of such an effect, we identified 13,786 genes of *M. musculus*, for which there were two isoforms represented in the mouse genome annotation. The sets of corresponding isoforms for the same gene were used separately as the references for aligning reads (Dryad **Table D7**). This analysis identified only a single gene, Gbp6, which encodes the interferon-inducible guanylate binding protein 6, as a DEG with FDR <0.05 that differed between isoform sets. In this case, it was RNA-seq with the second isoform set as the reference that missed this DEG call (**Supplementary Figure S2**). From this, we concluded that it was not likely that the greater number of isoforms identified for *M. musculus* than for *P. leucopus* substantively biased the analysis one way or the other.

By the gauge of fold changes of treated over control for paired cultures, the deermouse fibroblasts had a higher number of upregulated DEGs than what was recorded for the mouse cells (**Figure 2**). Nearly identical results were found at the 10-µg/mL exposure for *M. musculus* (**Supplementary Figure S3**), showing evidence that the lower number of upregulated DEGS in *M. musculus* was not likely attributable to greater resistance of mouse cells to the agonist. By this analysis, some genes of relevance to innate immunity that were upregulated in deermice cells but little if at all in mouse cells were Oxtr1 (oxytocin receptor) (32), Ebi3 (Epstein–Barr virus-induced gene 3) (33), and the protease inhibitor Wfdc12 (WAP four-disulfide core domain 12), which is homologous to Slpi (34). For *M. musculus*, two of the genes specifically upregulated in that species’ cells were Gpr84 (G protein-coupled receptor 84), which is a common loss-of-function allele among inbred strains mouse (35), and the gene for the cytokine interleukin-36A, which is known to be active in the skin (36).

By gene ontology (GO) term analysis of the DEGs for each species (**Supplementary Figure S4**), the responses of the deermouse and mouse cells were largely coherent, with the terms “innate immune response” (GO:0045087), “regulation of inflammatory response” (GO:0050727), and “inflammatory response” (GO:0006954) among the top 5 for each species and with *p*-values <10^−10^.

Further differences between the species’ fibroblast cultures were apparent when the DEGs were categorized according to specificity for one species or another, defined here as a ≥10× difference between species in respective fold changes for a given gene (**Table 1** **;** **Supplementary Tables S3**, **S4**). In the most discriminating cases, such as Pla2g2a (phospholipase A2, group IIA) and the PRR Pglyrp3 (peptidoglycan recognition protein 3) (37) of *P. leucopus* or Fth1 (ferritin heavy polypeptide 1) and the siderophore-binding Lcn2 (lipocalin 2) of *M. musculus*, the orthologous gene was not detectably transcribed in the comparison species. For some genes (e.g., Mx2 for *P. leucopus*), the distinctions between species were present under both control and treatment conditions, while for other genes (e.g., Il11 for *M. musculus*), the species difference was only noted for cells exposed to the agonist.

**Table 1:** Differentially transcribed coding sequences of genes between *Peromyscus leucopus* and *Mus musculus* second passage dermal fibroblasts under conditions of no treatment (control) or treatment with the TLR2 agonist Pam3CSK4 at 1 µg/mL.

Higher transcription in *Peromyscus* (P) than *Mus* (M)					Higher transcription in *Mus* than *Peromyscus*				
CDS	FC P/M cont.	P/M *t*-test *p* cont.	FC P/M Pam3CSK4	P/M *t*-test *p* Pam3CSK4	CDS	FC M/P cont.	M/P *t-*test *p* cont.	FC M/P Pam3CSK4	M/P *t-*test *p* Pam3CSK4
Arg1	14.6	6E−04	23.2	3E−04	Adamts9	2.20	2E−01	31.3	3E−04
Batf2	60.2	3E−06	113	4E−06	Aqp5	589	8E−09	73.9	2E−07
Bdkrb1	15.5	5E−07	14.8	4E−08	Arg2	5.65	2E−03	68.7	1E−05
Calhm5	4.56	2E−04	13.1	6E−07	Ass1	332	2E−06	1,032	6E−07
Casp1	12.8	7E−06	26.6	1E−06	Ch25h	0.66	1E−01	10.1	8E−07
Cd40	2.02	8E−02	13.1	7E−06	Chi3l1	80.5	2E−06	6.24	9E−03
Cmpk2	8.21	1E−03	25.8	8E−05	Cxcl11	2,862	2E-08	39.9	8E−04
Cxcl10	5.25	9E−04	12.6	7E−05	Cxcl5	15.7	5E−04	2.94	2E−04
Ddx60	13.3	5E−04	20.5	4E−06	Egln3	10.3	3E−03	11.8	4E−03
Dennd3	13.7	6E−06	48.4	3E−08	Ereg	28.8	3E−06	21.0	8E−05
Deptor	30.4	4E−06	27.2	6E−06	Fth1	43,525	1E−09	74,792	4E−10
Dpy19l2	14.6	4E−07	20.0	2E−07	Il11	1.92	6E−02	11.7	3E−05
Dtx3l	5.10	2E−06	16.2	2E−06	Il1rn	1,530	4E−07	84.0	2E−06
Hpx	3.35	2E−03	14.4	5E−04	Il36a	1.74	2E−01	142	6E−06
Icam4	0.96	9E−01	187	3E−10	Il4r	12.1	2E−06	2.70	1E−03
Ifi205	15.8	8E−06	41.8	1E−06	Il6	17.2	1E−05	1.75	1E−01
Il15	2.66	2E−03	18.6	1E−07	Ism1	4.09	5E−02	12.7	6E−04
Irf7	4.29	4E−03	13.2	5E−05	Lcn2	1,719	6E−07	12,898	8E−09
Irgm2	5.55	5E−05	43.7	6E−06	Lrig1	4.41	2E−04	20.8	3E−07
Kctd12	15.9	7E−09	17.4	2E−08	Lrrc17	90.5	3E−06	45.6	3E−06
Mmp12	4,372	9E−07	3,114	7E−07	Mmp3	11.1	4E−03	23.4	4E−04
Mx2	24.3	6E−05	66.9	5E−06	Mt1	18.9	1E−06	46.4	9E−08
Neurl3	5.31	2E−02	21.7	2E−04	Mt2	34.6	2E−05	20.6	9E−06
Oas1	10.3	2E−04	24.7	8E−06	Nos2	14.9	2E−03	26.1	3E−05
Pglyrp3	4.95	2E−02	1,243	2E−08	Rgs16	249	1E−06	640	6E−08
Pla2g2a	882	6E−08	222,063	5E−12	Saa3	172	2E−04	9.81	7E−06
Pla2g5	58.3	9E−07	281	7E−08	Sema7a	5.87	5E−03	12.0	5E−05
Plek	2.60	6E−02	18.4	8E−06	Serpina3f	13.7	2E−03	3.83	1E−02
Rcan1	9.90	1E−07	15.7	6E−07	Serpina3g	14.1	1E−03	4.98	3E−03
Rgs5	375	1E−06	364	2E−06	Serpina3i	37.1	2E−04	38.7	2E−04
Rnf122	1.68	5E−02	39.2	3E−08	Slco2a1	54.7	3E−04	123	5E−07
Rsad2	1.50	4E−01	23.0	7E−04	Slfn2	199	7E−11	683	7E−08
S100a8	15.5	4E−03	29.0	4E−05	Tnfsf11	1,465	3E−06	214	2E−07
Saa1	0.98	1E+00	57.7	7E−07	Tyk	7.50	5E−04	13.6	1E−03
Samd11	234	9E−11	97.8	3E−08	Wnt16	950	1E−07	164	6E−07
Selp	1.83	1E−01	78.3	1E−06					
Serpina3n	128	2E−05	1742	7E−06					
Slc2a6	2.99	3E−03	16.7	6E−08					
Slc7a2	14.7	3E−06	3.16	5E−03					
Sp110	104	3E−07	59.7	4E−08					
Srgn	0.45	4E−01	14.4	2E−04					
Stat4	323	2E−06	1,359	5E−08					
Tm4sf19	17,238	9E−12	6,108	7E−09					
Tnf	0.34	5E−03	19.3	1E−04					
Tnfaip6	4.85	2E−05	20.5	6E−08					
Tnfsf18	50.3	4E−04	781	2E−06					
Tnfsf4	12.3	4E−04	79.7	1E−05					
Traf1	2.57	3E−02	25.4	2E−07					
Trim30d	1,649	2E−07	2,293	1E−07					
Ubd	0.87	8E−01	148	1E−05					

The left side of the table has genes that had higher transcription in *P. leucopus* (P) than *M. musculus* (M), and the table’s right side has genes that had higher transcription in *M. musculus* than in *P. leucopus*. The targeted RNA-seq data (**Supplementary Table S2**) were normalized by total reads, adjusted for length as reads per kb, and then, for cross-species comparison, the ratio to Gapdh reads for the same sample. The five different cell cultures for each species (**Supplementary Table S1**) were analyzed as pairs within a species for both fold change (FC) and *t*-test under either control (cont.) conditions or Pam3CSK4 treatment.

Many of the genes or pathways that stand out in **Figure 2**, **Table 2**, **Supplementary Table S2**, or **Supplementary Figure S4** merit further analysis. But to do justice for all is beyond the scope of this report. Accordingly, we limit the focus to the following: genes that prior studies of experimental animals had shown had relevance for the phenomenon of infection tolerance demonstrated by *Peromyscus* species, and DEGs in this study that were unexpected.

**Table 2:** Differentially transcribed coding sequences (CDS) of genes between low-passage (P4) and high-passage (P47) dermal fibroblasts of *Peromyscus leucopus* from bulk RNA-seq analysis of fibroblast cells without or with exposure to Pam3CSK4 or LPS.

CDS name	CDS length (bp)	TPM group mean	Log_2_ fold change	FDR *p*-value	CDS name	CDS length (bp)	TPM group mean	Log_2_ fold change	FDR *p*-value
Rspo3	825	10	13.3	2E−08	Il6r	1,377	51	3.6	2E−75
Ly6e	411	104	11.2	1E−83	Irf9	1,278	38	3.6	3E−23
Vcam1	2,208	696	11.0	6E−48	Saa1	369	13	3.5	1E−05
Icam1	1,485	273	11.0	2E−32	Nfkbiz	2,205	34	3.5	6E−06
Tfpi2	705	80	10.7	3E−99	Irf7	1,401	77	3.0	3E−11
Cfb	2,301	376	10.7	6E−35	Cxcl1	300	1,396	2.8	8E−03
Ldoc1	423	243	10.6	3E−178	Eng	1,968	50	2.8	1E−89
Saa3	369	75	10.6	2E−17	Col6a2	3,081	363	2.8	9E−41
Pla2g2a	441	86	10.3	3E−19	Ccl7	462	1,599	2.8	5E−04
Cxcl5	498	102	10.2	6E−48	Nod1	2,814	76	2.7	5E−56
Csf3	627	110	10.2	7E−17	Ifih1	3,069	28	2.7	3E−15
Ifi203	2,709	38	10.1	1E−76	Stat2	2,853	38	2.6	4E−15
Adamts5	2,799	42	10.0	1E−89	Deptor	1,236	17	2.5	7E−13
Ndn	978	62	10.0	2E−143	Sting1	1,140	103	2.4	6E−22
Cxcl3	309	71	10.0	1E−20	Sod2	690	616	2.4	3E−06
Efemp1	1,479	240	10.0	1E−31	Ikbke	2,196	97	2.4	1E−14
Rsad2	1,083	23	9.9	2E−23	Ifngr1	1,404	163	2.3	1E−35
Prrx2	747	121	9.6	4E−64	H-2 Q10	1,206	127	2.3	2E−38
Sema3d	2,346	76	9.4	4E−27	Bst2	519	61	2.3	1E−06
Cxcl12	282	22	9.4	9E−34	Rigi	2,781	35	2.2	1E−06
Dpt	606	785	9.3	3E−63	H-2 DDa	1,086	105	2.2	4E−32
Col3a1	4,398	723	9.2	3E−67	Nfkb1	2,910	93	2.2	4E−10
Ago1	2,565	17	9.2	3E−150	H-2_LDa	1,215	216	2.2	5E−43
Col11a1	5,568	16	9.1	1E−79	Gbp2b	2,325	67	2.0	6E−03
Cd248	2,292	27	9.1	3E−226					
Dcn	1,092	688	9.1	7E−45	Tlr2	2,355	60	0.1	9E−01
Ednrb	1,329	14	9.0	2E−86	Tgfb1	1,173	128	−0.2	3E−01
Slpi	396	329	9.0	2E−74	Actb	1,128	6659	−0.3	2E−01
Lbp	1,446	89	8.9	3E−96					
Lum	1,017	50	8.8	2E−89	Insig1	774	127	−2.0	2E−32
Ccl27a	474	15	8.6	1E−46	Il11	1,092	15	−2.0	9E−15
Icam4	930	11	8.3	2E−16	Lpl	1,425	747	−2.1	2E−24
Plek	1,053	24	7.8	5E−93	Timeless	3,585	17	−2.1	2E−28
Col8a1	2,238	48	7.8	0E+00	Mmp2	1,986	22	−2.2	2E−28
Srgn	621	16	7.2	6E−17	Ung	1,305	27	−2.5	1E−34
S100b	279	13	7.0	2E−28	Brca1	5,469	21	−2.6	3E−55
Ctsk	990	174	6.8	0E+00	Myc	1,317	64	−2.6	3E−56
Il1r2	1,308	11	6.5	2E−95	Aqp1	810	698	−4.6	4E−88
Bdkrb1	996	93	6.3	1E−75	Col6a3	7,350	328	−4.7	2E−151
Col5a3	5,223	37	5.9	1E−58	Cxcl14	300	165	−5.1	3E−117
Irgm1	1,239	38	5.9	8E−17	Mmp9	2,157	13	−5.4	4E−16
Stat4	2,244	43	5.9	2E−188	Ca3	783	120	−5.8	2E−290
Igf1	462	105	5.6	5E−145	Abcg2	1,971	144	−6.7	0E+00
Il6	639	90	5.4	1E−14	Gzma	789	61	−6.8	1E−38
Sdc4	591	1,303	5.2	2E−24	Col18a1	3,972	65	−6.8	0E+00
Fscn1	1,482	185	5.1	2E−274	Sema7a	1,995	24	−7.3	4E−193
Cxcl11	306	133	4.8	2E−04	Il1rn	480	36	−7.4	2E−99
C4b	5,226	22	4.7	4E−213	Krt14	1,428	16	−8.0	4E−134
Rtp4	1,224	24	4.6	7E−17	Adamtsl2	2,775	89	−8.1	0E+00
H-2 DDa	738	71	4.2	4E−08	H19	351	3,554	−8.9	0E+00
Mx2	1,986	149	4.1	7E−16	Dkk2	780	133	−9.0	0E+00
C1r	2,118	778	4.0	3E−58	Col4a5	5,076	85	−9.8	0E+00
Col6a1	3,108	91	3.9	5E−182	Kpna7	1,503	133	−11.7	1E−239
Bcl3	1,344	41	3.9	6E−19	Cck	348	315	−13.1	3E−74
Tagln	606	195	3.7	2E−123					

The comparison was of the samples from three conditions for the P4 cells with the samples from the three conditions of the P47 cells. The table lists by columns the gene names, CDS lengths in base pairs (bp), the TPM mean, the log_2_ of fold change (FC) between the P4 cells compared to the P47 cells, and the false discovery rate (FDR) *p*-value. The genes comparatively upregulated in P4 cells are indicated by a yellow fill in the cells. The genes comparatively upregulated in P47 cells are indicated by blue fill. Selected examples of genes that were not differentially transcribed are indicated by no fill. The product names of the genes are provided in **Supplementary Table S3**. The data are drawn from the fuller roster of genes and the results of **Supplementary Table S7**.

### Nitric oxide and arginine metabolism pathways

A notable finding in prior studies of *P. leucopus* and *M. musculus* animals in response to LPS was a dichotomy between species in the blood and spleen in the relationship between the expression of nitric oxide synthase 2 (Nos2) and arginase 1 (Arg1) (19, 20). Deermice under treatment displayed a high Arg1-to-Nos2 ratio, which was consistent with the profile for alternatively activated (or M2) macrophages. The mice instead had an inverted ratio of Arg1-to-Nos2, which is more typical for classically activated macrophage (or M1) polarization (38, 39). The demonstration that LPS-treated, low-passage fibroblasts of *P. leucopus* did manifest transcription of Nos2 above baseline values indicated that this species had the capacity to express Nos2.

**Figure 3:**
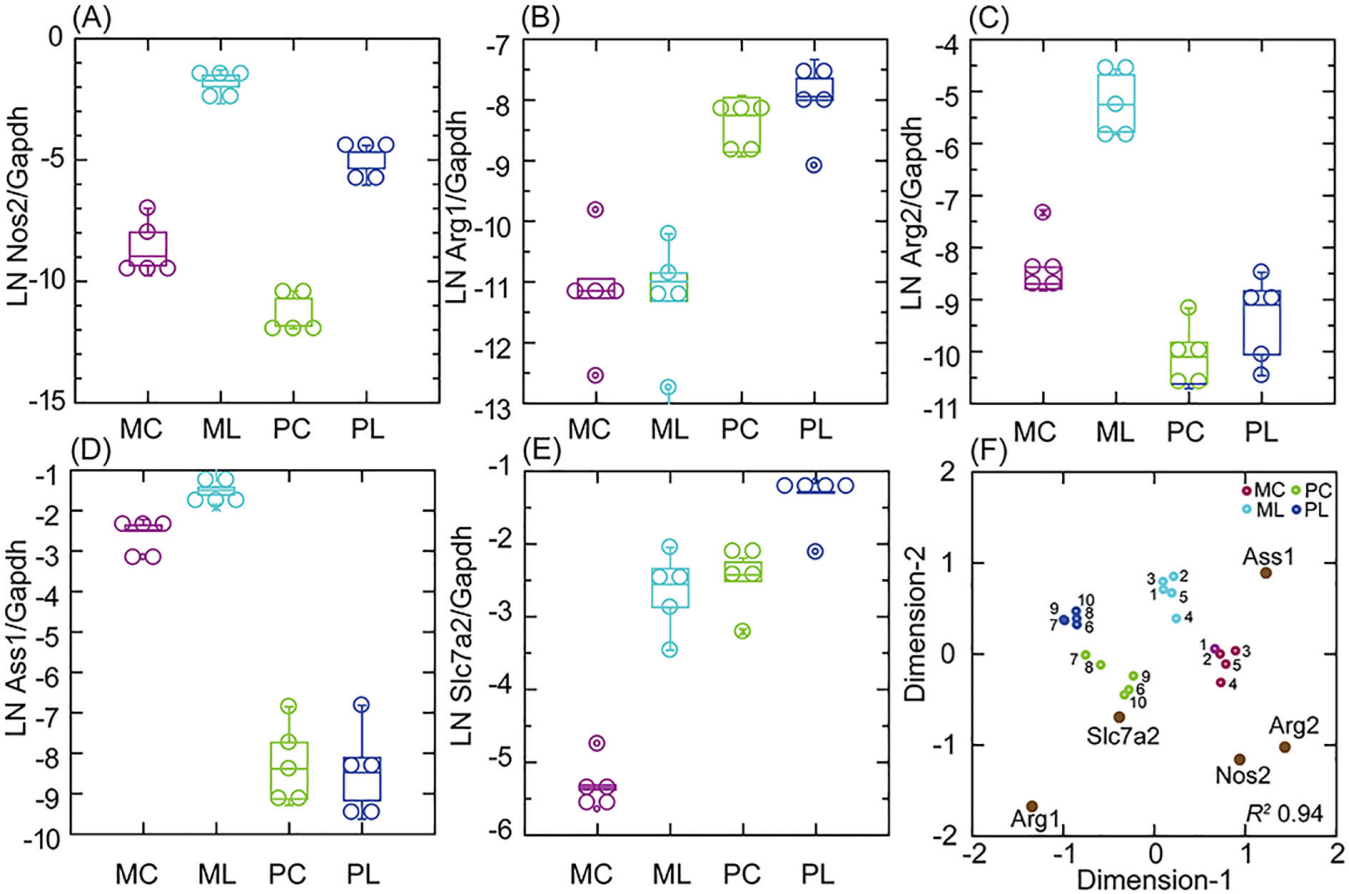
Transcription of nitric oxide and arginine metabolism CDS Nos2, Arg1, Arg2, Ass1, and Slc7a2 in targeted RNA-seq of primary dermal fibroblasts of *M. musculus* (M) or *P. leucopus* (P) without (C) or with (L) treatment with the lipopeptide Pam3CSK4 at 1 µg/mL. **(A–E)** Box–whisker plots of individual CDS. **(F)** A multidimensional scaling configuration plot of an analysis reduced to 2 dimensions (*x*- and *y*-axes) from the data for the box plots and with coordinates of CDS variables for the 10 samples indicated by gene names and symbols with dark brown fill. Pairs of individual lineages (1–5 for *P. leucopus* and 6–10 for *M. musculus*; **Supplementary Table S1**) are designated by numbers. The data for analyses and paired (within species) and unpaired (between species) *t-*test *p*-values are shown in **Supplementary Table S2**.

In the present study, we confirmed the transcription in *P. leucopus* cells of Nos2 by RNA-seq with a different set of dermal fibroblast cultures and with a different TLR agonist (**Figure 3**, **Supplementary Table S3**). The fold-change increase in Gapdh-normalized transcription of Nos2 between control and agonist-treated cells was similar in both species, but the expression of Nos2 was several-fold lower at baseline in the deermouse cells. This was accompanied by a high baseline transcription of Arg1 with modest further elevation with agonist exposure for the *P. leucopus* cells. While the *M. musculus* fibroblasts, like their cells in the blood, had low transcription of Arg1 under control and treatment conditions, transcription of Arg2, the gene for the mitochondrion-based type II arginase (40), was manyfold higher than observed for deermouse cells for both conditions (**Figures 2**, **3** **;** **Table 1**).

Of note were other arginine metabolism genes that in their profiles of transcription were distinguished between the species (**Figure 3**, **Table 1**). One of these was Ass1, the gene for arginosuccinate synthetase 1, which is also based in the mitochondria (41). Ass1 was little transcribed by *P. leucopus* cells but highly at baseline, and furthermore, with treatment in *M. musculus* cells. Next was Slc7a2, which encodes a cationic amino acid transporter for L-arginine (42). Like Arg2 in mouse cells, Slc7a2 transcription increased to a high level in agonist-treated cells. Unlike Arg2, which was little transcribed in *P. leucopus*, Slc7a2 was at baseline at the same normalized level as for the treated *M. musculus*. Taken together, Nos2, Arg1, Arg2, Ass1, and Slc7a2 could, in their multigene profiles, differentiate not only between species but also between conditions in each species (**Figure 3F**). The findings also point to differences between the metabolism of arginine and the extent to which this takes place in the mitochondria of cultured fibroblasts.

### Secretory leukocyte peptidase inhibitor

Another feature distinguishing *P. leucopus* from *M. musculus* after exposure to LPS was a more than 1,000-fold increase over baseline of transcription of Slpi in the blood, as documented by both RNA-seq and RT-qPCR. At the same time, there was only a marginal elevation in the treated mouse samples over the low levels observed for controls (19). A heightened expression of Slpi over baseline was also seen in *P. leucopus* infected with the relapsing fever agent *Borrelia hermsii*, as well as in a second experiment with LPS and with outbred mice (20). For LPS-treated cultures of ear skin fibroblasts, comparatively high transcription of Slpi was noted not just in the treated cells but in untreated cells as well (19).

In the present study, with an independent set of cultures from *P. leucopus* and which were processed in parallel with cultures from outbred *M. musculus*, we confirmed the high transcription of Slpi in untreated cells and, as before, with a modest increase after TLR2 agonist exposure (**Supplementary Table S3**). For this study, we also documented the expression in the *P. leucopus* fibroblasts of Slpi protein by MALDI-TOF. The following three peptides, which cover 46% of the 106 amino acid-processed protein (accession XP_028724460.1), were identified: KDSIKIGACPSISPAK, CTVPLPISRPVRRK, and KSGKCPTFQGRCMMLNPPNK.

The various pathways leading to Slpi expression are not well defined. To provide insight for this, we identified among 365 genes, which were upregulated DEGs for either or both *P. leucopus* and *M. musculus* (**Supplementary Table S2**), those that were highly correlated with Slpi transcription across species and conditions. The first transcription factor on the list of descending *R* ^2^ values was Nfe2l2, more commonly known as Nrf2, which encodes the protein nuclear factor, erythroid derived 2, like 2. Nfe2l2 is a transcriptional regulator of a number of genes involved in the adaptive response to oxidative and other cytotoxic stresses (43).

**Figure 4:**
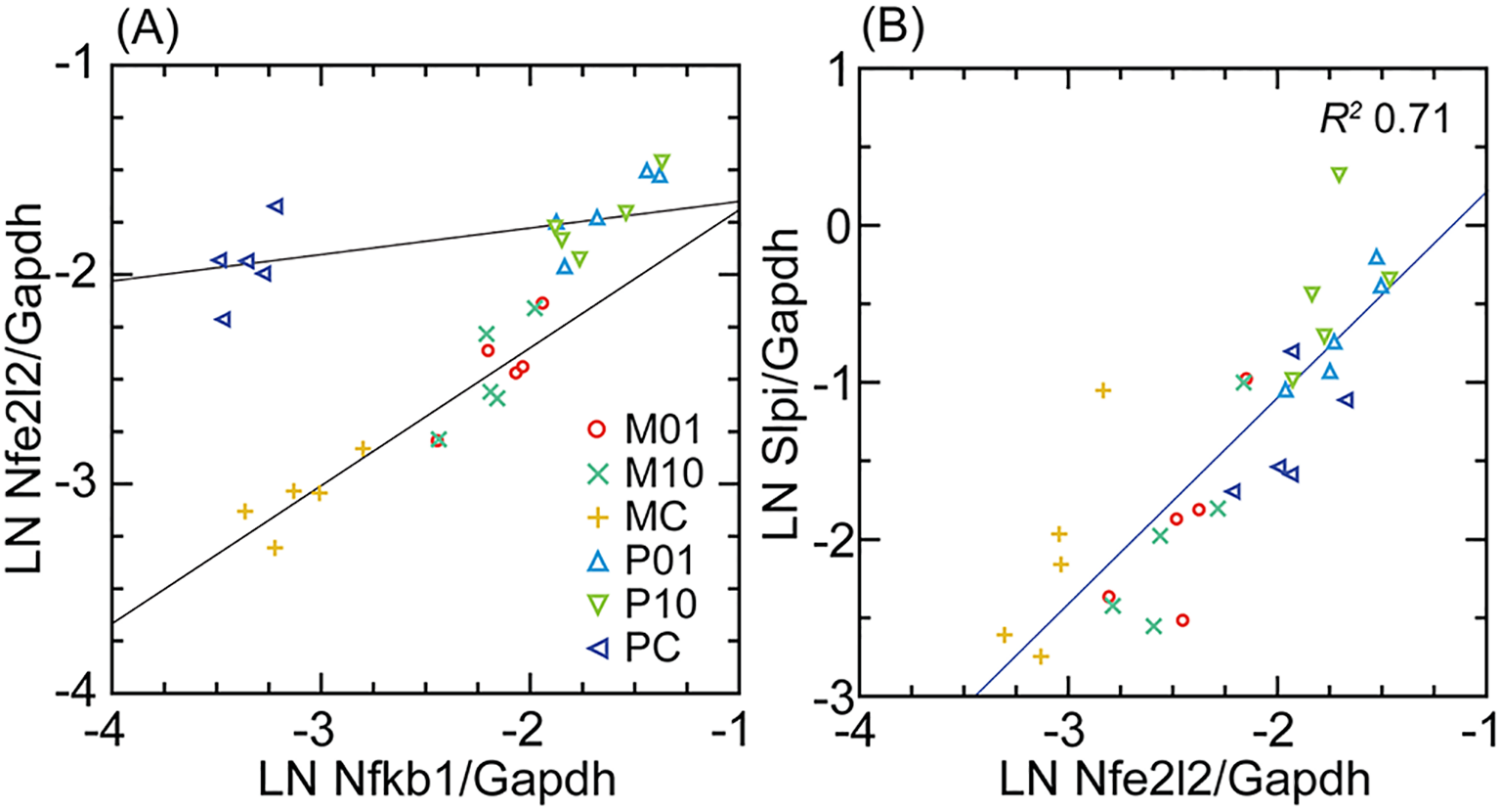
Scatter plots and linear regressions of normalized transcription of either Nfe2l2 (Nrf2) on Nfkb1 **(A)** or Slpi on Nfe2l2 **(B)** for *M. musculus* (M) and *P. leucopus* (P) dermal fibroblasts that either untreated (C) or treated with Pam3CSK4 lipopeptide (L) at 1 µg/mL (01) or 10 µg/mL (10). For analysis of **(A)**, the regression is separate for P and M. In **(B)**, the regression was for both species and with the coefficient of determination (*R^2^*) for both. Data for analyses are in **Supplementary Table S2**.

Nfe2l2 transcription in the deermouse and mouse fibroblasts was compared with that of Nfkb1, the gene for a more broadly active transcription factor (**Figure 4**). For the control cells, when Nfkb1 transcription was about the same in both species, Nfe2l2 expression was nearly as high for *P. leucopus* as it would be after exposure to the lipopeptide. While Nfe2l2 transcription in the treated *M. musculus* cells never reached the levels observed in *P. leucopus*, there was a still greater fold increase over baseline than was observed for deermouse cells. In contrast, the relationship between Nfe2l2 and Slpi was more direct and consistent across species and conditions, suggesting a regulatory role of Nfe2l2 in Slpi expression in *P. leucopus*.

### PRRs and interferon-stimulated genes

**Figure 5:**
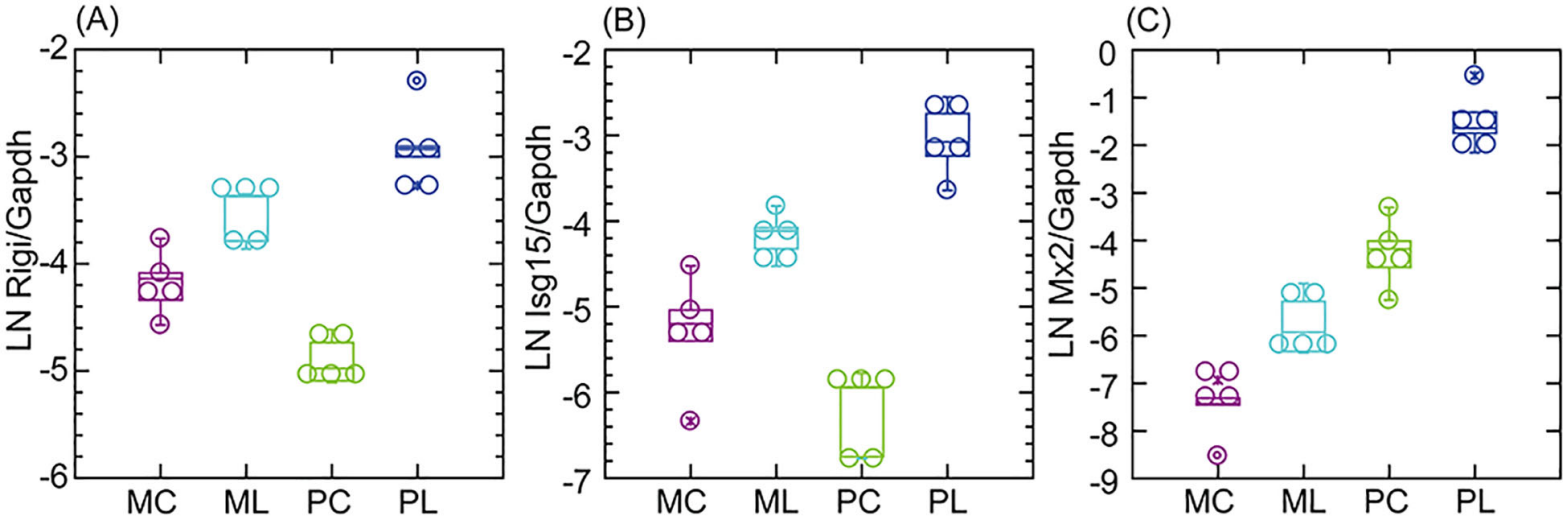
Box–whisker plots of log-transformed Gapdh-normalized transcription of the pattern recognition receptor (PRR) CDS Rigi **(A)** or the interferon-stimulated genes (ISG) Isg15 **(B)** or Mx2 **(C)** in *M. musculus* (M) or *P. leucopus* (P) dermal fibroblasts without (C) or with treatment with lipopeptide (L) Pam3CSK4 at 1 µg/mL. Data for analyses are in **Supplementary Table S2**.

The results with the fibroblast cultures replicated some of the previous findings with experimental animals. One observation for those PRRs and interferon-stimulated genes (ISGs) that were DEGs for either or both mouse and deermouse was that illustrated by the genes for the PRR RIG-I and Isg15 in panels (**A**) and (**B**) of **Figure 5, namely**, transcription that was lower in *P. leucopus* than *M. musculus* at baseline but then exceeded that of the mouse with agonist exposure. Another observed pattern is shown in panel (**C**). As for *P. leucopus* animals, the antiviral ISG Mx2 in the fibroblast cells was at an elevated level relative to the mouse at baseline and displayed an even higher level with treatment. Other genes demonstrating the second pattern in the fibroblasts, replicating the findings for the experimental animals, were the PRR Ifih1 (MDA5), the regulatory factor Irf7, and the antiviral ISG Oas1 (**Supplementary Figure S5**).

### Endogenous retrovirus and transposable elements

In a previous analysis of the blood of deermice and mice, we observed that the two species differed in their transcription of sequences for a retroviral envelope (Env) protein and Gag-pol polyprotein in response to the TLR4 agonist LPS (20). For the present study, we used much expanded reference sets of ~1 million annotated endogenous retrovirus (ERV) and derived transposable element (TE) sequences for each species (Dryad **Tables D4** and **D5**). The distribution of lengths of the ERVs in the complete sets was remarkably similar between species, with approximately equal proportions of sequences in different size classes, including those more than 5 kb in length (**Supplementary Figures S6A, B**). After exclusion of ~90% of the sequences with lengths under 500 bp, there remained 104,932 sequences for the *M. musculus* reference set and 103,397 for *P. leucopus* (Dryad **Table D6**). For these sets, the lengths ranged from 500 to 9,329 bp for *M. musculus* and from 500 to 9,543 bp for *P. leucopus*. The distributions in sequence lengths were skewed toward lower values and with a long tail to the right; the skewness statistic was 4.3 for mouse and 3.5 for deermouse. The median length (interquartile range) was 711 (572-950) bp for *M. musculus* and 742 (567–1,164) bp for *P. leucopus* (Dryad **Tables D8** and **D9**). For the control samples, mean values for the TPM-based transcription measure were 9.6 for *M. musculus* and 9.1 for *P. leucopus* sequences.

A closer look at the ERV loci with higher transcription levels in cells across all conditions revealed differences between species. If the sets of sequences were limited to the top 1,000 in descending order of mean TPM (i.e., the top ~1% for each species), the species were comparable in terms of the distributions of the length-adjusted TPM values (**Supplementary Figure S6C**). What distinguished the mouse sequences was their overall longer absolute lengths (**Supplementary Figure S6D**). The mean lengths were 2,622 (2,525–2,719) bp for mouse and 985 (942–1,029) bp for deermouse (*t*-test *p* < 10^−10^). The length difference was marked for the top 100 (i.e., ~0.1%) of transcribed sequences by TPM values; mean lengths for the top 0.1% were 3,019 (2,742–3,186) bp for mouse and 1,146 (972–1,319) for deermouse cultures.

**Figure 6:**
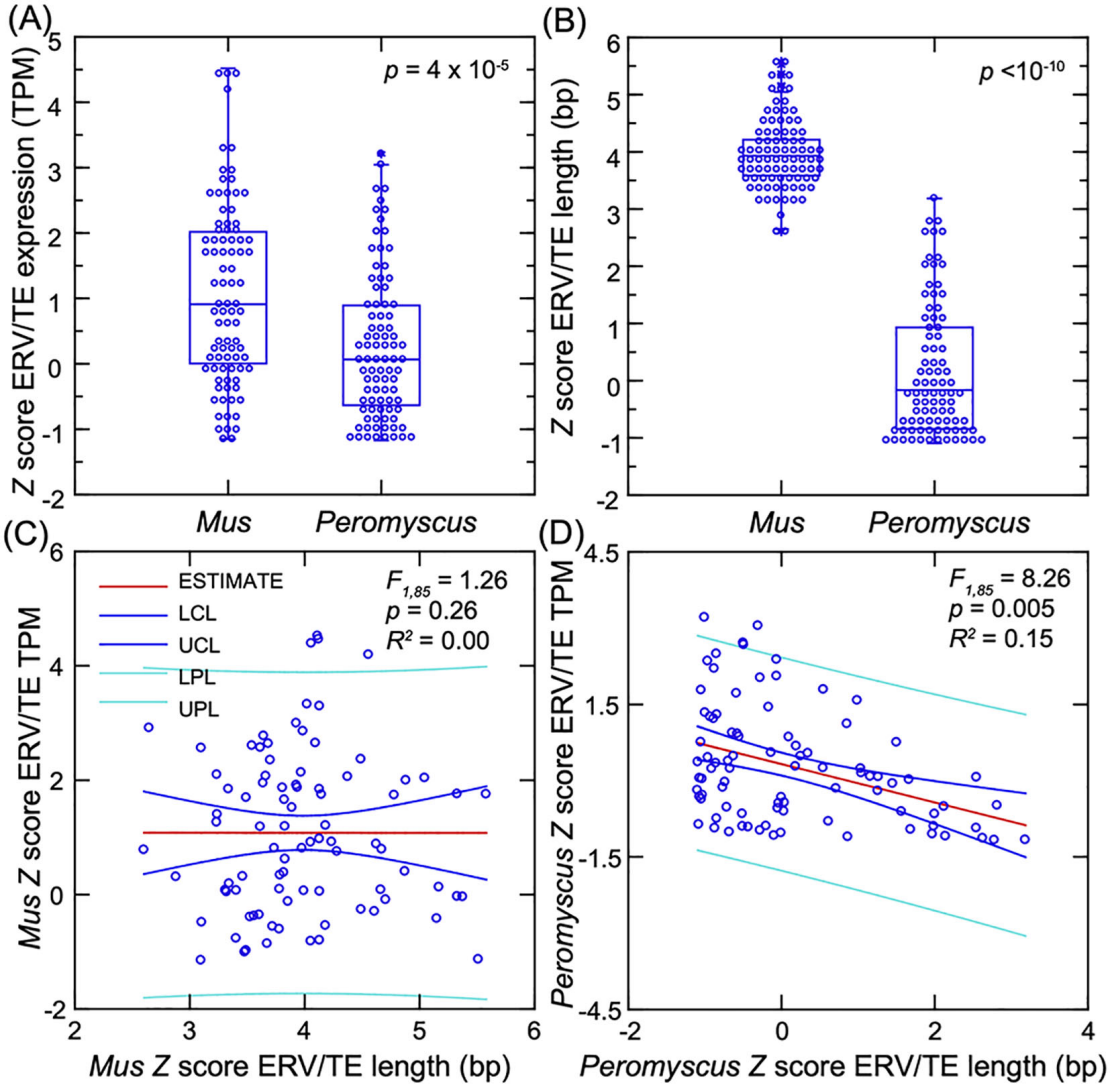
Comparison of the differentially expressed genes (DEGs) for ERV/TEs in five low-passage dermal fibroblast cultures each for *Mus musculus* and *Peromyscus leucopus* in the presence or absence of the TLR2 agonist Pam3CSK4. **(A)** Box–whisker plot of *Z*-scores of the mean TPM across the samples for each species for the 87 DEGs of *M. musculus* and 87 DEGs of *P. leucopus*. **(B)** Box–whisker plot of the *Z*-scores for lengths of the corresponding DEGs. The non-parametric test is the Kruskal–Wallis for the *p*-values of **(A, B)**. **(C, D)** General linear model regressions with estimates (mean), lower control limits (LCL), upper control limits (UCL), lower prediction limits (LPL), and upper prediction limits (UPL) for ERV/TE mean TPM on length for the DEGs of *M. musculus* **(C)** and DEGs of *P. leucopus* **(D)**. Data for analyses are from **Supplementary Table S4**.

We compared the five fibroblast lines each for *P. leucopus* and *M. musculus* for the fold-change differences in transcription between low-passage cells treated with 1 µg/mL of Pam3CSK4 and untreated cells. As we observed for the genome-wide CDSs (**Figure 2**), the number of DEGs with higher transcription after agonist exposure was greater than the number of DEGs with reduced transcription post-exposure for both species (**Supplementary Figures S6E, F**). There were subsets of 87 ERV/TEs each for *P. leucopus* and *M. musculus* that increased ≥2-fold in transcription in the presence of 1 µg/mL of lipopeptide and for which the paired *t*-test *p*-value was <0.001 (FDR < 0.05) (**Supplementary Table S4**). For cross-species comparisons, we used *Z*-scores for lengths, which were based on the distributions of lengths for the complete reference sets of ~100,000 sequences for each species, as well as Z-scores for mean TPM across all conditions, based on distributions of 5,296 mouse and 7,432 deermouse ERV/TEs with mean TPM ≥10 (**Figure 6**). Between these subsets, the mouse ERV/TEs tended to have higher mean transcription, but there was considerable overlap between species in the ranges for this measure (**Figure 6A**).

The characteristic more definitively distinguishing the species for these DEG subsets was length (**Figure 6B**). These *Z*-scores corresponded with ranges of lengths of 2,479–8,726 bp for *M. musculus* and 500–4,162 bp for *P. leucopus* (**Supplementary Table S4**). Not only were *P. leucopus* DEGs shorter in general than the mouse DEGs, but there was a trend of lower transcription with increasing length of the deermouse sequences that was not observed in mouse sequences (**Figures 6C, D**).

While there were no *P. leucopus* sequences in this subset longer than 4,162 bp, let alone 5,000 bp, 21 (24%) of 87 *M. musculus* upregulated ERV/TEs were ≥5,000 bp (*p* < 10^−10^). We identified in these 21 mouse sequences ORFs having an ATG as start codon (where A is position +1), that were at least 30 codons, and had both a purine at position −3 and a G at position +4. These are features of a Kozak sequence for a protein translation initiation site in vertebrates (44). They identify ORFs that are translated more plausibly than ORFs lacking these features. Of these 21 DEG ERV/TEs, 19 (90%) collectively had 115 ORFs meeting this criterion on either plus or minus strand and ranging in length from 30 to 1,091 amino acids and a mean of 92 (67-116) (**Supplementary Table S5**). Of these, 46 (39%) would encode all or part of an ERV Gag-pol polyprotein (*n* = 45) or Env protein (*n* = 1), in total constituting a mean proportion of 0.22 (range 0.06–0.44) of the lengths for 15 qualifying ERV/TEs. In contrast, for the 6 longest *P. leucopus* DEG ERV/TEs, which ranged from 3,034 to 4,162 bp (**Supplementary Table S5**), there were only 12 short ORFs (range of 93–258 bp) that met the Kozak sequence criteria, and only 1 of the predicted peptides of 31–86 amino acids had discernible similarities to either Gag-pol polyproteins or Env proteins of ERVs.

In sum, the mouse ERV/TEs that were upregulated in transcription in the dermal fibroblasts exposed to the TLR2 agonist were not only substantially longer than the DEG sequences for the deermouse fibroblasts, but there was also evident greater potential for translation of whole or parts of ERV/TE proteins than was noted for the deermouse DEGs among the ERV/TEs.

### Interleukin-11

Unlike preceding examples, which were of correspondences between the findings of *in vivo* and *in vitro* systems, some distinguishing genes for the fibroblasts would not have been predicted by studies of blood, spleen, or liver. One we highlight here is interleukin-11 (IL-11), the gene for which was an upregulated DEG in mouse fibroblasts, but lowly transcribed in deermouse fibroblasts, regardless of condition (**Table 1**). IL-11 is a member of the IL-6-type cytokine family; the specific receptor, IL-11Rα, is expressed by Il11ra in fibroblasts but not immune cells (45). An upstream transcription factor for IL-11 is NF-κB (Nfkb1). IL-11 is considered a key factor in the inflammation of aging (“inflammaging”) process, in part by promoting fibrosis (46, 47).

**Figure 7:**
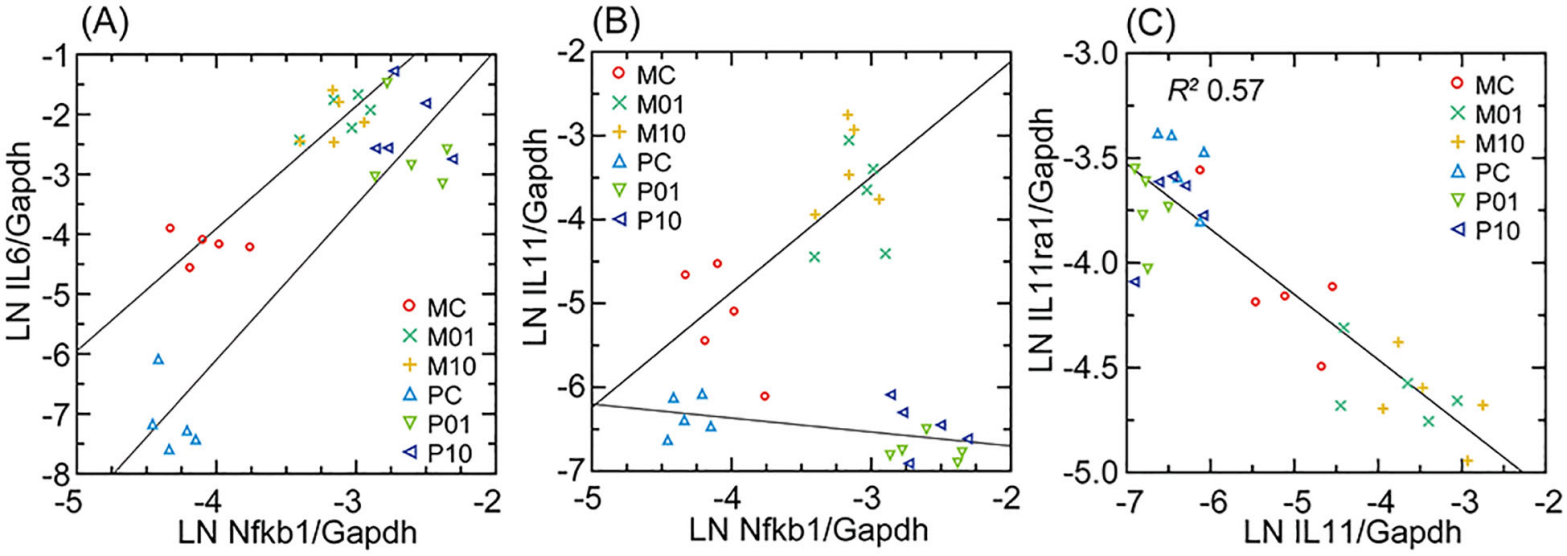
Interleukin-6 (Il6; **(A)**), interleukin-11 (Il11; **(B)**), and interleukin-11 receptor (Il11ra; **(C)**) Gapdh-normalized transcription compared in scatter plots and with linear regressions for *M. musculus* (M) and *P. leucopus* (P) dermal fibroblasts that were either untreated (C) or treated with Pam3CSK4 lipopeptide at 1 µg/mL (01) or 10 µg/mL (10). For analyses of **(A, B)**, linear regressions are separated by species. For **(C)**, the linear regression with the coefficient of determination (*R^2^*) was for both. Data for analyses are in **Supplementary Table S2**.

A targeted RNA-seq analysis of transcription of Nfkb1, Il6, Il11, and Il11ra, with Gapdh as the housekeeping gene for normalization across species, is shown in **Figure 7**. There were higher baseline levels of Il6 in mouse cells than deermouse cells, a greater magnitude of Il6 elevation with agonist exposure in deermouse cells, and similar Nfkb1 levels between species. The finding of the genome-wide RNA-seq analysis for Il11 was confirmed. While Il11 transcription levels increased in mouse cells with both concentrations of Pam3CYSK4, in deermouse, there was, if anything, a further decline from low levels at baseline. The scarce to absent Il11 transcripts in these low-passage *P. leucopus* cells were not accompanied by reduced expression of its receptor Il11ra, which was at comparatively high levels in deermouse control cells and, unlike mouse cells, did not decline with agonist exposure. These were the findings for the early passage cells of *P. leucopus*. As described below for high-passage cells, the capacity of *P. leucopus* for transcription of Il11 had not been lost.

### Single-cell RNA-seq of low-passage populations

At their outsets, the cultures of the full-thickness ear tissue included cartilage, capillaries and other small vessels, epidermis, hair, and hair follicles, as well as the dermis that would be the presumptive source for the fibroblasts (48). What emerged from this initial mixture of cell types were adherent cells for both *P. leucopus* and *M. musculus*. These could be released from the polystyrene dish bottoms with trypsin, and when these were used to populate a fresh culture, the cells soon became adherent again. They had the morphology of fibroblasts (**Figure 1**), and we had the bulk RNA-seq results, which, for both species, confirmed the expression of fibroblast markers, such as the collagen gene Col1a1, fibroblast-specific protein 1 (S100a4), and vimentin (Vim). However, heterogeneity was undefined.

As a first step to better characterize these primary cultures, we carried out single-cell RNA-seq of two sets of pooled cells, one for each species. Each pool contained equal parts of cells that were untreated, cells exposed to Pam3CSK4 for 4 h, and cells exposed to LPS for 4 h. The reasoning was to represent, in a single population, cells after exposure to two different TLR agonists, as well as cells at baseline, and then processed them together. Each species’ sample yielded ~4 × 10^4^ cells and, after downstream processing, indexing, library preparation, and sequencing, ~10^9^ PE150 reads.

**Figure 8:**
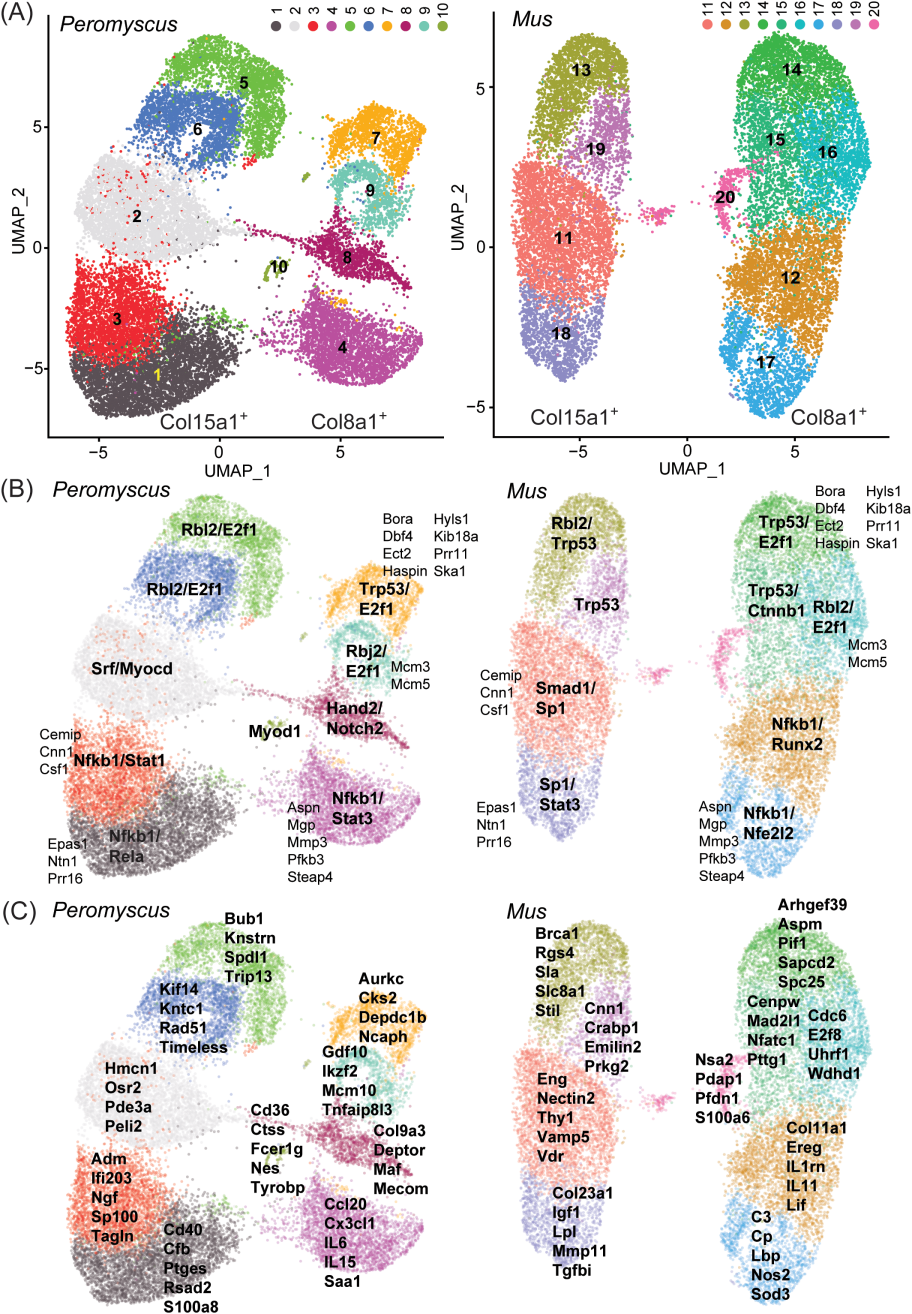
Single-cell RNA-seq of low-passage-cultivated dermal fibroblasts of *Peromyscus leucopus* and *Mus musculus* with 2-dimensional UMAP projections of clusters of types of cells in pooled populations of cells that were untreated or exposed to either Pam3CSK4 or LPS. **(A)** The clusters of each species are identified by number and color: 1–10 for *P. leucopus* and 11–20 for *M. musculus*. Two CDS that served as markers for the left and right superclusters were Col15a1 (left) and Col8a1 (right). **(B)** Transcription factors that were predicted by GO term analysis to be associated with signifying CDS for each cluster (**Supplementary Table S6**) are indicated in bold font. CDS that defined the same clusters in both species are in a smaller and regular font. **(C)** CDS that were enriched in representation in the clusters and that discriminated to that degree between species are listed for each cluster.

We approached the analysis without set ideas about markers to prioritize. Gene expression matrices were normalized and dimensionally reduced by principal component analysis. UMAP projections and cell clustering were generated by the top principal components for each cell. There were 10 major clusters for each species (**Figure 8A**), numbered 1–10 for *P. leucopus* and 11–20 for *M. musculus*. For both species’ cells, these were organized in two superclusters, shown on the left and right in each graphic. The individual clusters for each species were roughly in the same spatial arrangement, with the exceptions of cluster 10 of *P. leucopus* and cluster 20 of *M. musculus*, which did not have clear counterparts in the comparison species. By analyzing single genes, we found that the transcription of two collagen genes, Col15a1 and Col8a1, specifically distinguished between left and right superclusters in both species, thus providing evidence of comparability between deermouse and mouse cultures in terms of cell type representation at this higher order.

Provisional cross-species analogies among clusters were supported by predictions of what transcription factors would be predominant from GO term analysis of the genes contributing to cluster discrimination (**Figure 8B**, **Supplementary Table S6**). Transcription factors associated more generally with normal cell growth, such as E2f1 and Rbl2, appeared to define the upper clusters (i.e., 5, 6, 7, and 9 of *P. leucopus* and 13–16 and 19 of *M. musculus*) of each of the left and right superclusters of deermouse and mouse. On the other hand, the lower poles of both superclusters were associated with transcription factors, such as Nfkb1, Nfe2l2 (Nrf2), Stat1, and Stat3, more typically identified with host responses to a stressor, suggesting to us that these lower pole clusters were the cells exposed to one or the other TLR agonist. For half of the clusters, there were also between 2 and 8 genes that contributed to defining a cluster in the same relative location in both deermouse and mouse. These were clusters 1, 3, 4, 7, and 9 for *P. leucopus* and 11, 14, and 16–18 for *M. musculus*. These included the Epas1 (endothelial PAS domain protein 1) for clusters 3 and 11 and Csf1 (colony-stimulating factor 1, macrophage) for clusters 1 and 11.

If only the 4–5 top-ranked genes by degree of enrichment and that uniquely contributed to cluster identity were considered (**Figure 8C**), we observed again in the lower halves of the two superclusters a predominance of genes that were associated with innate immune and other host responses. Of note, with regard to other aspects of this study, are the antiviral effector Rsad2 and calgranulin component S100a8 in cluster 1, interleukin-6 and the chemokine Cxcl20 in cluster 4, interleukin-11 and the interleukin-1 receptor antagonist Il1rn in cluster 11, and Nos2 and complement component C3 in cluster 17. The small cluster 10 located between the two superclusters of *P. leucopus* stood out from the other 9 clusters of the deermouse fibroblasts and all 10 mouse clusters in the specifying contributions of dendritic cell-associated genes Cd36, Fcer1g (CD23), and Tyrobp (DAP12). Cluster 20 of *M. musculus* was defined in part by the genes listed in the panel (C) of the figure, but also by enrichment of lower magnitude for several ribosomal proteins (**Supplementary Table S6**), an indication that this was a more rapidly dividing cell population, without a counterpart in the *P. leucopus* fibroblast sample.

### Spontaneously transformed *Peromyscus leucopus* fibroblasts

Bulk RNA-seq was carried out on cultures of *P. leucopus* at passage 4 (P4) and passage 47 (P47), which were controls or had been treated with Pam3CSK4 or LPS for 4 h. The number of PE150 reads ranged between 2.13 and 2.93 × 10^8^ with a mean of 2.45 × 10^8^. The primary objective was to identify genes that were transcribed in P4 cells but to little or no degree in P47 cells or vice versa. For this purpose, we compared the three samples of P4 against the three samples of P47 cells across conditions. By the criterion of a fold change of ≥4, mean TPM ≥10 across all samples for a given gene, and an FDR *p*-value <0.01, 80 genes were differentially upregulated in P4 cells and 25 were upregulated (denoted by negative fold-change values) in P47 cells (**Tables 2**, **S7**). Genes that were little transcribed in P47 compared to P4 cells were Col8a1, Deptor, Eng, Il6, and Lbp, each of which was more closely associated with the right-side superclusters in the single-cell experiment (**Figure 8**). On the other hand, upregulated genes in the transformed cells included Il1rn, Il11, and Timeless, which were associated with the left supercluster. This indicated that the lineage of transformed cells can be traced to the left supercluster fibroblasts than the fibroblasts on the right.

Besides the near-complete or complete loss of expression of several of the innate immunity-associated transcription factors, cytokines, chemokines, and ISGs that featured in the comparison with low-passage mouse fibroblasts, there was also diminished transcription of MHC class I protein genes in P47 cells. In addition to the upregulated Il11 and Il1rn, which were notably lowly transcribed in early passage deermouse cells in comparison to mouse cells (**Table 1**), two cancer-associated genes, Brca1 and Myc, were more highly transcribed in P47 than in P4 cells.

A secondary objective was to identify in the transformed cell line those genes that not only were still transcribed but also increased in expression after exposure to either or both of the TLR agonists (**Supplementary Table S8**). If this were the case, it would indicate retention of some signaling pathways of cell-autonomous immunity. One of these was Slpi, which was 1,000-fold lower in transcription in P47 cells than in P4 cells across all conditions (**Table 2**), but still was 10-fold higher in transcription in the agonist-treated P47 cells than in the controls. Another was Cxcl1, which was 100-fold higher in transcription in Pam3CSK4-treated P47 cells than in the controls. The chemokine Cxcl10 was not one of those diminished in transcription in P47 versus P4 cells, but, like Cxcl1, it displayed greater than 10-fold upregulation in transcription with the TLR2 agonist but not the TLR4 agonist.

The reads were also aligned with the *P. leucopus* reference set of 103,397 ERVs of ≥500 bp. Across the six samples, 10,843 (10.5%) ERV/TEs had a mean TPM ≥5. Of these, 832 (7.7%) of ERV/TE sequences were more highly transcribed in low-passage than in high-passage cells across all three conditions by the criterion of FDR *p* < 0.01 and absolute fold change ≥2 for three samples for each passage history (**Supplementary Table S9**). By the same criteria, 1,136 (10.5%) were more highly transcribed in high-passage cells (denoted in the table as negative fold-change values).

The distinguishing DEGs of P47 cells were of interest because of their association with an ostensibly immortalized state. The high-passage cells’ upregulated DEGs were, in general, longer (mean of 1,189 vs. 1,082 bp; *p* = 0.01) and more highly transcribed (mean TPM of 328 vs. 81; *p* = 0.003) than low-passage cell DEGs. Of the 14 high-passage cell DEGs of ≥5 kb size, 3 were >9 kb, with lengths (and chromosome locations) of 9,543 (NC_051064.1:6812577-6822120), 9,432 (NC_051076.1:33708849-33718281), and 9,208 (NC_051063.1:54636550-54645758) bp. Not only do the three have ORFs for components of Gag-pol polyproteins, including reverse transcriptases, but they also would encode retroviral Env proteins. For the ERVs of 9,543 and 9,432 bp, the CDS for Env sequences aligned with feline leukemia virus (FLV) proteins along their full lengths and without frame shifts or in-frame stop codons. The Env CDSs of the 9,543- and 9,432-bp ERV sequences have a purine at the −3 position and ATGG for positions +1–4, thus plausibly translatable. The 1,983-nt sequence of the predominant FLV-related Env polyprotein mRNA (GenBank accession PV892740) transcribed by the transformed *P. leucopus* fibroblasts would encode a polypeptide of 661 aa with a gp70 portion from positions 32 to 463 and the more conserved, transmembrane p15E portion from positions 464 to 638.

In contrast, the six DEGs of ≥5 kb, fold change ≥2, and FDR *p* < 0.01 of low-passage cells were of a maximum length of 7,503 bp (**Supplementary Table S9**). The six ERV sequences each comprised ORFs for parts of Gag-pol polyproteins, but none would discernibly encode a retroviral envelope protein, in part or whole. This and other findings about the immortalized fibroblasts showed that amidst the silence of many genes of inflammation consequence, such as Il6, other genes, such as some ERV sequences and Il11, were effectively released from repression.

## Discussion

Previous comparisons of *P. leucopus* with *M. musculus* used laboratory-bred animals that were euthanized at the end of the experiment (19, 20, 49, 50). Acknowledging that *in vivo* studies deserve their preeminence in research, we asked whether pre-mortem primary cultures of a tissue from *P. leucopus* could stand in for the animals themselves for some research questions. For field-based studies, this would allow for the release of captured animals after measurements and specimens have been taken. This was one rationale for the application of wing tissue biopsies for studies of bats in nature (51, 52). For laboratory-based investigations of stock colonies, longitudinal designs with non-lethal sampling of tissue at different times become feasible.

An indisputable advantage of tissue culture is the capability to freeze the cells indefinitely, permitting replication of an experiment or the application of procedures not available at the time of collection. With their tissues propagated and cultures split, animals can serve as their own controls in experiments. There can be interventions that could not be feasibly tested with live animals because of toxicity at the organismal level or impeded delivery to the tissue or organ of interest. Experiments on cultured cells do not suffer from these restrictions. These manipulations include RNAi and other non-transgenic gene silencing methods or CRISPR gene editing applications, before this technology is achieved for the whole animal.

These are justifications for the use of cultured cells; however, their validity as approximations of live animals rests on two assumptions. The first is that cells cultured from a non-model organism, e.g., *P. leucopus*, sufficiently resemble those of a more established model organism, i.e., the house mouse, that one can exploit extant databases and literature on the model organisms. If there are many incongruities or misalignments, the findings for the deermouse might be limited in impact to that one genus. The second assumption is that *in vitro* experiments’ results generally line up with those of *in vivo* experiments for the same organism. In other words, if a gene is upregulated in the animal under a certain condition, is it also upregulated in the tissue culture cells?

In considering the first assumption, we noted the report that established cultures of *P. leucopus* fibroblasts had similar doubling times to those of *M. musculus* (53). Unlike cultured fibroblasts of humans and some other large mammals, deermouse and mouse cells had telomerase activity and did not undergo replicative senescence in the study of Seluanov et al. (53). The observed decline in the present study of one *M. musculus* culture within a few passages is provisionally attributed to greater oxygen sensitivity of the mouse cells (29) and not replicative senescence. Without discounting a possible distinction between species in their responses to oxidative stress, the early passage populations of *P. leucopus* and *M. musculus* dermal fibroblasts had more in common than differences by genome-wide RNA-seq (**Supplementary Figure S1** **;** **Figures 2**, **3**).

With acknowledgment that analogies between scRNA-seq UMAP clusters for different species should be made with caution, we propose that the assignment of correspondences between species was justified not only based on their locations in the maps but also by signifying genes in common (**Figure 7** **;** **Supplementary Table S6**). Col15a1 and Col8a1 empirically distinguished the left and right superclusters of both deermouse and mouse in this experiment (**Figure 7**), but these genes are not generally recognized as markers for the predominant types of fibroblasts in mouse skin: papillary fibroblasts in the upper dermis and reticular fibroblasts in the lower dermis (54). Some markers known for their specificities for fibroblast types in intact skin would be expected to decline in expression in culture (55), thus reducing the number of informative characteristics available for cell origin typing. Nevertheless, some enrichments of expression allowed for tentative assignment of origins for some clusters. Examples were Crabp1 (cellular retinoic acid binding protein 1) and Col23a1 for left-sided clusters and Aspn (asporin) and Col11a1 for right-sided clusters (**Supplementary Table S6**). These suggest to us that the left and right superclusters had origins in papillary fibroblasts and reticular fibroblasts of the dermis, respectively, of mouse and deermouse.

Justifying the second assumption is a challenge, considering differences in complexity and time scale *in vivo* and *in vitro*. But, as noted by Tyshkovskiy et al. (56), who cited examples from research on aging, a rationale for this assumption exists. One of those examples was the report of Ma et al. (57). These investigators cultivated fibroblasts from the skin of captured *P. leucopus* and *P. maniculatus*, along with several other small- and medium-sized mammals, and found that the RNA-seq and biochemical assay results with *in vitro*-cultivated cells generally conformed with known longevity traits of the animals themselves. We similarly noted correspondences between prior findings for blood, spleen, and liver from deermice and mice and the findings with cultivated dermal fibroblasts of those species in experiments in which the time frames for the exposures to the agonists were similar. Whether this would also hold true for shorter or longer periods of exposure remains to be determined.

One of these correspondences was Slpi, which encodes an inhibitor of proteases of neutrophils and other phagocytic cells. Among its functions, the secreted protein has a role in wound repair (58) and has an inhibitory effect on the formation of neutrophil extracellular traps (59). Of relevance for a study of an infection-tolerant reservoir for the Lyme disease agent was the report that Slpi-deficient mice infected with *B. burgdorferi* had more severe arthritis and greater bacterial burdens in their joints than their wild-type counterparts (60). In prior studies, we observed that expression of Slpi increased orders of magnitude in the blood of *P. leucopus* treated with LPS or infected with a relapsing fever *Borrelia* species (19). For LPS-treated mice, there was only a modest increase in Slpi transcription from a low level. These observations notwithstanding, it was not given that dermal fibroblasts of *P. leucopus* would transcribe Slpi and produce the protein, but that was the finding here. Transcription was high at baseline in the deermouse cultures and increased further with TLR2 agonist exposures (**Figure 4**). In contrast, with the mouse cells, there was the same comparatively low expression of Slpi as observed in the *in vivo* experiment. While there remain questions about the regulation of Slpi expression, the tight correlation of Slpi transcription across species with that of the stress-responsive transcription factor Nfe2l2, more commonly known as Nrf2, indicates a direct regulatory role, as has been reported for the mouse (61). Nfe2l2 regulates the transcription of a variety of cytoprotective proteins, including antioxidants, such as heme oxygenase 1 and superoxide dismutase 2, and chaperones (62). **Figure 4** reveals a high constitutive level of transcription of Nfe2l2 by the *P. leucopus* fibroblasts compared to the control *M. musculus* cells. This is a characteristic of the long-lived naked mole rat, an established experimental model for aging research (63), and the comparatively long-lived Snell dwarf mutant mouse (64). Constitutive expression of Nfe2l2 has also been associated with longevity in the nematode *Caenorhabditis elegans* (65). A possible explanation for the successful serial propagation of the deermouse cells, but not the mouse cells, in the presence of atmospheric concentrations of oxygen was a greater capacity to withstand oxidative stress.

Another distinguishing feature of *P. leucopus* cells of the blood and spleen was the near absence of transcription of Nos2 (19, 20), which encodes the mitochondrion-based, inducible nitric oxide synthase. This was when Arg1 was transcribed at high levels at baseline and even higher with LPS exposure. As noted previously for *P. leucopus* fibroblasts (19) and in the present study (**Figure 3**), Nos2 is transcribed constitutively and at higher levels after exposure to an agonist of TLR4 or TLR2. But this was starting at a level manyfold lower than for the *M. musculus* fibroblasts at baseline. Arg1 expression was hardly detectable in the mouse fibroblasts and substantially higher in deermouse cells under both control and treated conditions, similar to what was observed in the blood and spleen. What the *in vitro* study added to the picture of arginine metabolism in the two species was the differential expression of two other arginine metabolism genes associated with the mitochondrion, Arg2 and Ass1, and an arginine transporter, Slc7a2 (42), which, unlike Arg2 and Ass1, was transcribed at comparatively high levels both in controls and agonist-treated deermouse cells (**Figure 3**). These findings draw attention to the mitochondria and the dichotomy between *P. leucopus* and *M. musculus* in the sites of activity for arginine metabolism genes of recognized importance in immunity (66, 67).

As in the study of whole blood of *P. leucopus* and *M. musculus*, there were differences between species in the transcription of ERV and TE sequences in cultured fibroblasts. In the study of LPS-treated animals, this was restricted to selected retroviral envelope protein sequences, which decreased in transcription in deermice while increasing in mice (20). In the present study, much larger sets of the ERV sequences of small to large size were used as references. These datasets were comparable between species in numbers and in the overall distributions of lengths and over the wide ranges of levels of transcription. Given the high redundancy of ERVs of all sizes in the mammalian genomes, confidently ascribing a location for the source of a ~150-nt cDNA read is a challenge, and this limitation, which applies to all studies of this sort, is acknowledged. That is why the emphasis was on the lengths of the ERVs as a class and not their precise identification. With that disclaimer, we found that the more highly expressed ERV sequences at baseline were generally longer in *M. musculus* than in *P. leucopus* (**Supplementary Figure S6**) and that this was also true for those sequences that were upregulated DEGs for one species or another (**Figure 7**).

Deermice have recently experienced massive invasions of diverse ERVs, resulting in a large expansion of KRAB zinc finger proteins (KRAB-ZFPs) responsible for ERV suppression (27, 68). This expansion of suppression machinery could enable deermice to control ERV expression more effectively, with important implications for inflammation, immune response, and longevity (69–71). Consistent with this, previous work has shown evidence that *P. leucopus* may control ERV expression more effectively compared to other rodents (20). Our data here also generally support this interpretation. However, we also observe expression of full-length ERVs in *P. leucopus*, encoding all or most of the proteins of a leukemia-type retrovirus. Interestingly, this particular ERV recently arose in the deermouse germline via interspecies horizontal gene transfer, exists at low copy numbers, and encodes full-length env genes that are expressed, suggesting that it might still function as an infectious virus (27). Such ERVs often evade host suppression machinery, so this ERV’s expression might not be too surprising. Nonetheless, these observations lead to important questions about the dynamics of ERV suppression in deermice and how more effective ERV suppression might impact immune response to other pathogens.

A more manageable number of ERV sequences to consider were the upregulated DEGs unique to transformed *P. leucopus* cells. Among the P47 DEGs were three ERVs of over 9 kb and which would encode Env proteins as well as Gag-pol polyproteins. Activation of RNA tumor viruses in spontaneously transformed mouse cell lines with or without inciting factors has long been noted (31, 72). *Peromyscus leucopus* provides another example of this, but this phenomenon in this species remains incompletely characterized. A future step would be documentation of the expression of viral proteins by these longer ERVs, such as with specific antibodies, mass spectrometry, or an assay for reverse transcriptase activity. While the P47 fibroblasts behaved as if immortal, including increased expression of the oncogene Myc (**Table 2**), other distinguishing features of transformed cells, such as their karyotypes, have not been examined.

In distinction to these parallels between *in vitro* and *in vivo* results, an *in vitro* finding lacking a counterpart in animal studies was a difference between species in transcription of the cytokine interleukin-11. In the blood, spleen, or liver of mice and deermice, there was scant to no transcription of Il11 under either baseline or agonist exposure conditions (19, 20). Does the low transcription of Il11 in primary cultures of deermouse fibroblasts fairly represent what occurs in other tissues or other pathologic conditions? Re-examination of results of RNA-seq of the skin of *P. leucopus* with or without infection with *B. burgdorferi* found no detectable transcription of Il11 (6). Whether there is an association of IL-11 with aging in *Peromyscus*, as has been reported for other species (46, 47), remains to be determined.

What we can consider here is the possible source of the Il11 transcription in the fibroblasts. This was evident for the mouse fibroblasts: the right-sided cluster 12 (**Figure 7**). Leukemia inhibitory factor (Lif), another member of the IL-6-type cytokine family, and interleukin 1 receptor antagonist (Il1rn) were also associated with cluster 12 (**Supplementary Table S6**). Il11, Il1rn, and Lif were more highly transcribed in P47 than in P4 deermouse cells (**Tables 2**, **S7**) and, unlike some chemokines, like Ccl2 and Cxcl10, were unchanged in transcription in P47 cells exposed to Pam3CSK4 or LPS (**Supplementary Table S8**). This suggests that, with spontaneous transformation and possible dedifferentiation, there was a release of repression of these genes, allowing for constitutive expression. The pro-inflammatory cytokine IL-6 also had right-side associations, namely, cluster 4 of *P. leucopus* (**Figure 7**). However, unlike the other IL-6-type family members, Il6 transcription was much decreased in the transformed cells (**Table 2**), an indication of a different form of regulation.

As the distinguishing features between *P. leucopus* and *M. musculus* mount and the list lengthens, a relationship between the infection-tolerant phenotype and the greater longevity of this deermouse begins to emerge. Similar differences between deermouse and house mouse had also been noted in comparative studies with different experimental designs for either the animals themselves (73) or bone marrow-derived macrophages (74). We knew before of the low susceptibility of *P. leucopus* to what would be lethal doses of LPS for a mouse, the moderated expression of interferons and downstream genes, and a polarization profile of alternatively activated macrophages tilting toward an anti-inflammatory outcome. The present study adds these distinctions: divergent natural histories of cultured fibroblasts, constitutive expression of the stressor-response transcription factor Nfe2l2/Nrf2, diminished expression of the aging-associated cytokine IL-11, and an indisposition to transcribe long ERVs with their potential to elicit recognition by PRRs and the inflammatory consequences of that.

Can we identify ramifications of the work of particular relevance to Lyme disease, as manifested either in an experimental animal model or human illnesses as we encounter them? After all, the agonist chosen for the study is one of the identified PAMPs of *B. burgdorferi* and other Lyme disease agents of the genus. Of particular interest would be findings from the present study that might point to new lines of investigation for Lyme disease research. We think one of these is the phenomenon of widespread transcription of sequences of ERVs and TEs, both under baseline conditions and differentially after exposure to the lipopeptide PAMP in mouse and deermouse cells. The conjecture is not that there is production of whole virions; there is no evidence of that in either our studies or elsewhere in the literature. Rather, it is that translated products, such as Env protein fragments or parts of reverse transcriptases, of retrovirus-derived sequences serve as PAMPs for cell-autonomous immune responses that would not be expected for infections by bacteria that are largely extracellular in the host (20). Two other topics that, in our view, merit further study in experimental models and in clinical research studies of Lyme disease are immunity-associated genes of arginine metabolism (66, 67) and mitigations against oxidative stress, especially by genes under the influence of the transcription factor Nrf2/Nfe2l2 (62).

## Materials and methods

### Animals

The study was carried out in accordance with the recommendations in the National Institutes of Health’s *Guide for the Care and Use of Laboratory Animals*: Eighth Edition of the National Academy of Sciences, and according to ARRIVE guidelines (https://arriveguidelines.org). The University of California Irvine (UCI) protocols AUP-23–127 and AUP-24–042 were approved by UCI’s Institutional Animal Care and Use Committee.

*Peromyscus leucopus*, here also referred to as “deermice,” were of the outbred LL stock that originated with 38 animals captured near Linville, NC, and thereafter comprised a closed colony without sib-sib matings at the Peromyscus Genetic Stock Center at the University of South Carolina (75). Deermice used in this study were each from different mating pairs and bred at the UCI vivarium. Outbred *Mus musculus* strain CD-1 and inbred *M. musculus* FVB/NJ strain, here also referred to as “mice,” were obtained from Charles River Laboratories and Jackson Laboratory, respectively. Rodents were maintained in the AAALAC-accredited UCI vivarium, with two to five animals per cage, a 16-h light/8-h dark schedule, room temperatures of 21 °C–23 °C, and humidity of 30%–70%. Food and water were available *ad libitum*; the diet was 2020X Soy Protein-Free Extruded Rodent Diets (Teklad, Madison, United States). The rodents were euthanized by carbon dioxide overdose followed by exsanguination by open thorax cardiac puncture.

### Tissue culture medium

The medium was RPMI 1640 medium (Gibco, Grand Island, United States) supplemented with 10% heat-inactivated fetal bovine serum (Gibco), 2 mM of L-glutamine, 100 µM of L-asparagine, 50 µM of 2-mercaptoethanol, 250 ng/mL of amphotericin B (Gibco, Fisher Scientific), 500 U/mL of penicillin G, and 500 µg/mL of streptomycin (76).

### Processing of tissue

Immediately after euthanasia, ~1 cm diameter of full-thickness ear tissue was excised with sterile instruments, immersed in 70% ethanol for 5 min, and allowed to dry in a biosafety cabinet. Once dry, the tissue was transferred to a 1.8-mL polystyrene screwcap tube containing 1.5 mL of medium with 2 mg/mL of collagenase type I (Millipore Sigma, Burlington, United States). Ear tissue was minced while in the tube with scissors down to ~3 mm size, and then the suspension was incubated at 37 °C on a horizontal shaker at 200 rpm for 75 min. The suspension was then pushed through a 70-µm cell strainer with the rubber end of a 5-mL syringe plunger into 10 mL of medium. The pass-through liquid was centrifuged at 400×*g* at room temperature for 5 min. The supernatant was removed, the pellet was suspended in 10 mL of medium, the suspension was centrifuged again, and the supernatant was removed.

### Tissue cultivation

After the final centrifugation, the cell pellet was suspended in 10 mL of pre-warmed medium. This was transferred to a 10-cm-diameter polystyrene culture dish (Fisher Scientific FB0875712) with lid and incubated at 37 °C in a humidified atmosphere with 5% CO_2_ (passage 0). After 24 h, the medium was removed by aspiration, adherent cells were washed with phosphate-buffered saline, pH 7.4 (PBS) once, and then the fresh medium was added at that point and then every 3–4 days as needed. The cultures were monitored by phase microscopy at ×100 magnification with an Olympus CK2 inverted phase contrast microscope. For subcultures (passage 1 onward), when the adherent cells were 80%–90% confluent, the medium was aspirated, and the cells were washed once with PBS. To release the cells, 2 mL of a solution of 0.02 mM of trypsin and 0.48 mM of sodium EDTA (trypsin-EDTA) was added before a 5-min incubation. The resultant suspension of cells was transferred to a 15-mL polystyrene conical tube containing 8 mL of medium and centrifuged at 400×*g* for 5 min. The supernatant was removed, and the cell pellet was resuspended in 10 mL of supplemented medium. A 1:1 ratio of 0.4% w/v trypan blue in PBS and cell suspension was used for manual counting of cells with a Petroff-Hausser counting chamber under the inverted microscope at ×200 magnification. Once counted, 100,000 cells were seeded into a new 10-cm petri dish with 10 mL of pre-warmed medium for an initial density of 10,000 cells/mL at time 0. The remaining cell suspension was centrifuged at 400×*g* for 5 min. The cell pellet was resuspended in 1 mL of medium with 10% cell culture-grade dimethyl sulfoxide and then transferred to 2-mL screwcap cryogenic vials (Greiner, Kremsmünster, Austria) and stored in liquid nitrogen. For determination of population doubling time, the cultures were monitored daily until adherent cells were 80%–90% confluent. After trypsinization, the harvested cells were suspended in 10 mL of medium, and counts of cells per mL were made at time *x*, where *x* is the interval in days from time 0. The log_10_ of the densities at times 0 and *x* were plotted against time for a log-linear regression.

### Photomicrographs

Digital images were taken on an Olympus CK2 inverted phase contrast microscope equipped with a LabCam Ultra (iDu Optics, Detroit, United States) adapter for an iPhone 14 Pro (Apple Computer) and with the ×15 magnification eyepiece, ×10 magnification objective lens, and ×2 digital zoom. The final magnification was ×150 for the 4,032 × 3,024 resolution High-Efficiency Image File Format (HEIF) format files.

### Chemicals

The lipopeptide Pam3CysSerLys4 (Pam3CSK4; Invivogen, San Diego, United States) and ion-exchange chromatography-purified lipopolysaccharide (LPS) of *Escherichia coli* O111:B4 (Sigma-Aldrich, Burlington, United States) were dissolved as stock solutions at 1 mg/mL concentration in endotoxin-free, sterile 0.9% (w/v) NaCl (saline; Sigma-Aldrich). Stock solutions were stored at −20°C in a non-frost-free freezer until the day of use, and dilutions were freshly prepared in saline on the day of the experiment.

### Cell culture treatments

Cell cultures at the point of 80%–90% confluency were treated with trypsin-EDTA, counted using a hemocytometer, and split into three wells each in a six-well polystyrene tissue culture plate (Fisher Scientific) at 3 × 10^5^ cells per well containing 2 mL of medium. For the study of single cells, there were 5 × 10^5^ cells per well. Cells were incubated at 37°C in 5% CO_2_ for 24 h prior to treatment, before either saline alone or Pam3CSK4 or LPS in saline was added for final concentrations of 1 or 10 µg/mL for Pam3CSK4 or 1 µg/mL for LPS. The suspensions were incubated at 37°C in 5% CO_2_ for 4 h, which is the interval in the previous *in vivo* experiments between injection of the TLR agonist and termination (19, 20).

### RNA extraction

After incubation with the TLR agonist or medium alone, the medium was aspirated, and 300 µL of DNA/RNA Shield (Zymo Research, Irvine, United States) was added to adherent cells, followed by the addition of 300 µL of Zymo RNA Lysis Buffer (Zymo Research). The Quick-RNA Miniprep Plus Kit (Zymo Research) was used for the isolation of skin fibroblast RNA following the manufacturer’s instructions, including DNase treatment. RNA was eluted from the spin column in 50 µL of Zymo Nuclease Free Water. Quantification, purity, and RNA integrity were assessed using a High Sensitivity Qubit 2.0 fluorometer, a Nanodrop ND-1000 spectrophotometer, and an Agilent Bioanalyzer 2100. The RNA integrity number (RIN) values for the samples were ≥9.0.

### Bulk RNA-seq

cDNA libraries were produced with the Illumina TruSeq Stranded mRNA kit. Multiplexed libraries were sequenced at UCI’s Genomics Research and Technology Hub on an Illumina NovaSeq 6000 with paired-end chemistry, 150 cycles, and ~120–250 million reads per sample. Read quality was analyzed by FastQC (https://www.bioinformatics.babraham.ac.uk/projects/fastqc/). Fastq files of reads were trimmed of low-quality reads (Phred score of <15), adapter sequences, and homopolymeric 5’ or 3’ ends using Trimmomatic (77). Trimmed reads were aligned to reference sets with a length fraction of 0.35, a similarity fraction of 0.9, and a cost of 3 (out of 3 maximum) each for a mismatch, insertion, or deletion to the reference sets using CLC Genomics Workbench v. 25 (Qiagen Aarhus A/S). Library size normalization was done by the TMM (trimmed mean of M values) method (78).

For the 22,654 *P. leucopus* protein-coding sequences (CDS), manual annotation of 1,141 gene loci without assigned gene names in the GCF_004664715.2 assembly and annotation (out of a total of 7,148 with “LOCxxxxxxxxx” designations) had been carried out on an *ad hoc* basis in those cases when a locus was identified as transcribed and a candidate DEG by RNA-seq in the present or a previous study (79). For the 22,760 protein-coding genes identified in the *M. musculus* C57BL/6 reference genome transcripts (GCF_000001635.27_GRCm39), there were 97,760 isoforms. For 3,927 loci, there was a single listed isoform, 13,786 loci with two isoforms, and 6,047 with three or more. For comparability with the *P. leucopus* dataset, the first listed isoform in the *M. musculus* reference genome CDS dataset was used (80). To assess whether other isoforms besides the first listed for *M. musculus* would provide additional or different results by RNA-seq, the 13,786 CDS with two listed isoforms were used. The sequences for these were extracted as separate sets of isoform 1 and isoform 2 for each of these CDS (Dryad **Table D7**) and subsequently used for independent RNA-seq and differential gene expression analysis.

For fold-change (FC) analyses of results that paired individual *P. leucopus* (*n* = 22,654 CDS) or *M. musculus* (*n* = 22,760 CDS) cell lines, unique counts were first normalized by total reads for the corresponding sample across the species and then adjusted as reads per kilobase (Dryad **Tables D1** and **D2**). Paired *t*-tests were performed with pairing of the same cell line under different conditions. For cross-species comparisons of the 14,979 CDS in common and with assigned gene names (Dryad **Table D3**), the ratio of length-adjusted, total count-normalized reads for a given gene to the housekeeping gene Gapdh was used. The mean (standard deviation) log_10_ Gapdh normalized unique counts were 4.8 (0.05) across 15 P*. leucopus* samples (i.e., 5 cell lines × 3 conditions) and 5.1 (0.05) across 15 *M musculus* samples. There was little or no discernible effect of the treatments on the transcription of Gapdh: the mean (standard deviation) paired fold changes (paired *t*-test *p*-value) of Pam3CSK4-treated cells to control were 1.08 (0.16), 1.12 (0.08), 1.00 (0.90), and 1.13 (0.07) for the mouse 1 µg/mL, mouse 10 µg/mL, deermouse 1 µg/mL, and deermouse 10 µg/mL sets, respectively (Dryad **Table D3**). Following the recommendation of Hedges et al., we used the natural logarithm (LN) of ratios (81).

For the input data for gene ontology term analysis (see below), differential gene expression (DEG) within a species and across conditions (i.e., without pairing by cell line), transcripts per million (TPM) were used as the expression level. The DEG analysis was conducted using the CLC Genomics Workbench suite’s Differential Expression Analysis tool, which is similar to EdgeR and DESeq2 in assuming a negative binomial distribution for expression levels and fits a separate generalized linear model for each (82). There is an adjustment for dispersion (76), and for this analysis, it was without downweighting of outliers.

### Gene ontology term analysis

The analysis was implemented with the tools of Metascape (https://metascape.org) (83) with default settings and *M. musculus* as the closest taxon for comparison. Similarity matrices were hierarchically clustered, and a 0.3 similarity threshold was applied to trim resultant trees into separate clusters. The lowest *p*-value term represented each cluster shown in the heatmaps. Besides the terms beginning with “GO” of the Gene Ontology resource (http://geneontology.org) (84), other terms refer to the KEGG Pathway database (https://www.kegg.jp) for “mmu.” designations, WikiPathways database (https://www.wikipathways.org) for “WP…” designations, and Reactome database (https://reactome.org) for “R-MMU…” designations.

### Transposable element sequences

We annotated ERV and other TEs in both the *M. musculus* C57BL/6 and *P. leucopus* genomes using a combined non-redundant database of lineage-specific and ancestral TEs in each species. To do this, we first retrieved curated TE models for *P. maniculatus* and *M. musculus* from DFAM (https://doi.org/10.1186/s13100-020-00230-y), as well as all ancestral TE models for each species. Then, we clustered redundant models using CD-HIT-EST (version 4.8.1) (https://doi.org/10.1093/bioinformatics/bts565) with these parameters: -n 10 -c.8 -r 1 -i. To annotate TEs in each genome using our combined TE library as input, we employed RepeatMasker (version 4.1.2) (https://www.repeatmasker.org) with these parameters: -pa 12 -excln -s -no_is -u -noisy -html -xm -a -xsmall. The names, genome locations, and lengths for the identified ERVs and TEs are provided in Dryad **Table D5** for *M. musculus* and **Table D6** for *P. leucopus*. For bulk RNA-seq analysis with these sequences as the reference sets, the measure of expression was TPM instead of unique reads. We wrote a custom python script (Supplementary_Text_3) to identify in these subsets of ERV sequences those ORFs that had an ATG as start codon (where A is position +1), were at least 30 codons, and had both a purine at position −3 and a G at position +4. The identified protein products of the ORFs meeting these criteria were used for BLASTP searches (https://blast.ncbi.nlm.nih.gov/Blast.cgi) with default settings of non-redundant protein sequences for *M. musculus* and gammaretroviruses of the GenBank database (https://www.ncbi.nlm.nih.gov/genbank/).

### Single-cell RNA-seq

After incubation of 5 × 10^5^ second passage *P. leucopus* fibroblast cells or second passage *M. musculus* strain FVB/NJ fibroblast cells with medium and saline alone, medium with 10 µg/mL of Pam3CSK4, or medium with 1 µg/mL of LPS, the medium was aspirated from the wells. The source animals were female and ~220 days of age. Adherent cells were washed with 2 mL of PBS. A 0.5-mL volume of trypsin-EDTA solution was added to each well, and the cells were incubated at 37°C in 5% CO_2_ for 5 min. Detached cells from each condition were pooled into 15-mL polystyrene conical tubes by species, and 10 mL of medium was added to neutralize trypsin activity. The pooled cell suspensions for *P. leucopus* and *M. musculus* were centrifuged at 400×*g* for 5 min, and the supernatant was removed. Cells of pellets were resuspended in Illumina Cell Suspension Buffer of the Single Cell 3′ RNA Prep Kit (Illumina). Cell viability and counts were determined in triplicate using trypan blue stain and a hemocytometer counting chamber. Cells were diluted in cell suspension buffer to a concentration of 5,000 per µL concentration, and a final total count was determined. Approximately 40,000 cells for each species were further processed using an Illumina Single Cell 3′ RNA Prep Kit for mRNA capture, cDNA synthesis, and library preparation, according to the manufacturer’s instructions. cDNA quality control and fragment analysis were performed using a DNA High Sensitivity chip on an Agilent BioAnalyzer 2100. A post-library preparation quality control, fragment analysis, and KAPA library quantification (Roche, Indianapolis, United States) by quantitative polymerase chain reaction were performed. cDNA libraries were sequenced on an Illumina NovaSeq X Plus system with paired-end reads, 150 cycles, and incorporation of a 2% PhiX spike-in. The yields were 1.6 × 10^9^ and 1.8 × 10^9^ reads for *M. musculus* and *P. leucopus*, respectively.

### Single-cell RNA-seq analysis

The raw fastq files were processed using DRAGEN v 4.4.2 software on the BaseSpace Sequencing Hub (Illumina). The reference genomes used were the default GCF_000001635.20 genome on the site for *M. musculus* and an imported genome (GCF_004664715.2) for *P. leucopus*. At the data analysis stage for the *P. leucopus* reference set, updated annotations with formal gene names (80) substituted for loci with incomplete descriptions in the original annotation. The DRAGEN output included filtered matrix, features, and barcode files, which were subsequently analyzed in *R* (85) using Seurat v5.2.1 (https://satijalab.org/seurat) (86). Seurat facilitated Uniform Manifold Approximation and Projection (UMAP) clustering via principal component analysis (PCA), visualization of gene expression on UMAPs through feature plots, cluster modifications, and identification of the enriched genes for each cluster. For the UMAP, principal components were used with the Seurat FindNeighbors setting from 1:10 and the FindClusters resolution set to 0.5. For the single-cell RNA-seq data, the criteria for GO term analysis of signifying genes of clusters were an enrichment of ≥1.5 and *p*-value <10^−4^ (**Supplementary Table S6**).

### MALDI-TOF mass spectrometry

After aspiration of the medium, adherent *P. leucopus* dermal fibroblasts of low or high passage grown on 10-cm-diameter polystyrene petri dishes were washed with 5 mL of PBS. Adherent cells were lysed by the addition of 1 mL of radioimmunoprecipitation assay buffer with protease inhibitor (Thermo Fisher). The petri dish bottom was scraped with a cell scraper and homogenized. Cell lysate suspension was transferred to a sterile 1.5 mL polystyrene screwcap tube, and sonication was performed with alternating cycles of 15 s of sonication and 15 s on ice over 1 min. Lysates were then centrifuged at 21,000×*g* for 10 min at room temperature, and the supernatant was decanted. The lysate was subjected to sodium dodecyl sulfate polyacrylamide gel electrophoresis on a 4%–12% gradient Bis-Tris denaturing polyacrylamide gel on a NuPAGE apparatus (Invitrogen, Waltham, United States). Electrophoresis was performed, and Coomassie blue-stained bands of the desired size range were excised. In-gel digestion with trypsin was carried out as described by Shevchenko et al. (87). In brief, destaining was performed with a 1:1 acetonitrile and ammonium bicarbonate, followed by reduction with dithiothreitol and alkalization with iodoacetamide. An overnight trypsin digestion at 37 °C was carried out, and this was followed by the extraction of the mixture of peptides, spotting them onto a plate reader, and air drying. Cell analytes were measured using matrix-assisted laser desorption/ionization (MALDI) on a Bruker UltrafleXtreme instrument at the UCI Department of Chemistry Mass Spectrometry Facility. The ribosomal protein S18 (Rps18; XP_028744221.1) and its known amino acid sequence served as a control for the targeted analysis of the secretory leukocyte peptidase inhibitor (Slpi). The reference for Slpi was the processed protein (amino acids 26–131) after cleavage of the signal peptide (XP_028724460.1). Spectra were acquired using a mass range from 800 to 6,000 Da. The FlexControl software v. 4.0 (Bruker) was used for data collection, and raw spectral data were imported into mMASS v. 5.5 (https://github.com/xxao/mMass) (88). Baseline correction and peak picking were performed using mMASS default settings.

### Statistics

Unless otherwise stated, means are presented with 95% confidence intervals in parentheses. For data that were not normally distributed, these were with asymmetrical confidence intervals. Parametric (*t*-test) and non-parametric (Kruskal–Wallis) tests of significance were two-tailed. Unless otherwise stated, the paired *t*-test *p*-value is given. Categorical variables were assessed by a two-tailed Fisher’s exact test. False discovery rate (FDR) correction of *p*-values for multiple testing was by the Benjamini–Hochberg method (89), as implemented with the False Discovery Rate Online Calculator (https://tools.carbocation.com/FDR). Linear regression, coefficient of determination (*R^2^*), multidimensional scaling (MDS), and general linear model (GLM) analyses were performed with the SYSTAT v. 13.1 software (Systat Software, Inc.). Box plots with whiskers display the minimum, first quartile, median, third quartile, and maximum.

### Data resources

**Supplementary Table S1** includes links to BioProject and BioSample numbers for samples of *P. leucopus* and *M. musculus*, along with accession numbers for raw reads (SRA) and RNA-seq analysis (GEO). The low- and high-passage *P. leucopus* dermal fibroblast study is registered under BioProject PRJNA1169383 with BioSamples SAMN45236449-54, SRA accessions SRR31668268-73, and GEO accessions GSM9100082-87. The single-cell RNA-seq study is registered under BioProject PRJNA1277016 with BioSamples SAMN49093172 for *P. leucopus* and SAMN49093173 for *M. musculus* dermal fibroblasts. The corresponding SRA accessions were SRR33982422 and SRR33982421, respectively. For this study, the 5′ UTR and coding sequence for the interleukin-11 gene Il11 of *P. leucopus* LL stock was determined by sequencing of mRNA using the RNA-seq reads. This was deposited at GenBank under accession PV639634; it corrects an error in the predicted protein-coding sequence in the NCBI automated annotation for accession XM_037199381.1. Tables of large datasets deposited with the Dryad (https://datadryad.org) public repository and denoted in the text by the convention “Dryad Table Dx” are accessible at https://doi.org/10.5061/dryad.m905qfvdq. Descriptions of Dryad datasets are in the Supplementary Materials (Supplementary_Text_2) and in references (79) and (80).

## Data Availability

The datasets presented in this study can be found in online repositories. The names of the repository/repositories and accession number(s) can be found in the article/**Supplementary Material**.
