# Supplementary material for: Dermal fibroblast cultures recapitulate differences between deermice and mice in their responses to a Toll-like receptor agonist
Source: Front Immunol. 2025 Nov 4;16:1666789. doi: 10.3389/fimmu.2025.1666789 (PMC12623179; doi:10.3389/fimmu.2025.1666789)
Supplement: Supplementary file 1 [file DataSheet1.docx]

Supplementary Text 1: This file with list of contents

Supplementary Text 2: List of large dataset files at Dryad repository

Supplementary Text 3: Python script
