# Supplementary material for: Dermal fibroblast cultures recapitulate differences between deermice and mice in their responses to a Toll-like receptor agonist
Source: Front Immunol. 2025 Nov 4;16:1666789. doi: 10.3389/fimmu.2025.1666789 (PMC12623179; doi:10.3389/fimmu.2025.1666789)
Supplement: Supplementary file 3 [file DataSheet3.docx]

python process_fasta.py in.fa out.csv

# process_fasta.py

import csv

import sys

from Bio import SeqIO

from Bio.Seq import Seq

import re

def find_orfs(seq, min_length=30):

orfs = []

pattern = re.compile(r'([AG][ACGT][ACGT]ATG(?:(?!TAA|TAG|TGA)...)*(?:TAA|TAG|TGA))')

for strand, nuc in [(+1, seq), (-1, seq.reverse_complement())]:

for match in pattern.finditer(str(nuc)):

start = match.start()

end = match.end()

orf = nuc[start:end]

if len(orf) >= min_length * 3 and orf[6] == 'G': # Check if the 7th base is G

if strand == -1:

start, end = len(seq) - end, len(seq) - start

orfs.append((strand, start, end, orf))

return orfs

def process_fasta(file_path, output_path):

with open(output_path, 'w', newline='') as out_file:

csv_writer = csv.writer(out_file)

# Write header

csv_writer.writerow(["transcript", "strand", "start", "end", "length", "peptide"])

for record in SeqIO.parse(file_path, "fasta"):

seq = record.seq

orfs = find_orfs(seq)

for strand, start, end, orf in orfs:

# Adjust start and end positions to exclude the 3 upstream bases

adjusted_start = start + 3

adjusted_end = end

# Adjust the ORF sequence to exclude the 3 upstream bases

adjusted_orf = orf[3:]

peptide = adjusted_orf.translate()

csv_writer.writerow([

record.id,

'+' if strand == 1 else '-',

adjusted_start + 1, # +1 because biological coordinates are 1-based

adjusted_end,

len(adjusted_orf),

str(peptide)

])

if __name__ == "__main__":

if len(sys.argv) != 3:

print("Usage: python process_fasta.py <input_fasta> <output_file>")

sys.exit(1)

input_file = sys.argv[1]

output_file = sys.argv[2]

process_fasta(input_file, output_file)

print(f"Results have been written to {output_file}")
