# Supplementary material for: Dermal fibroblast cultures recapitulate differences between deermice and mice in their responses to a Toll-like receptor agonist
Source: Front Immunol. 2025 Nov 4;16:1666789. doi: 10.3389/fimmu.2025.1666789 (PMC12623179; doi:10.3389/fimmu.2025.1666789)
Supplement: Supplementary file 4 [file Image1.pdf]

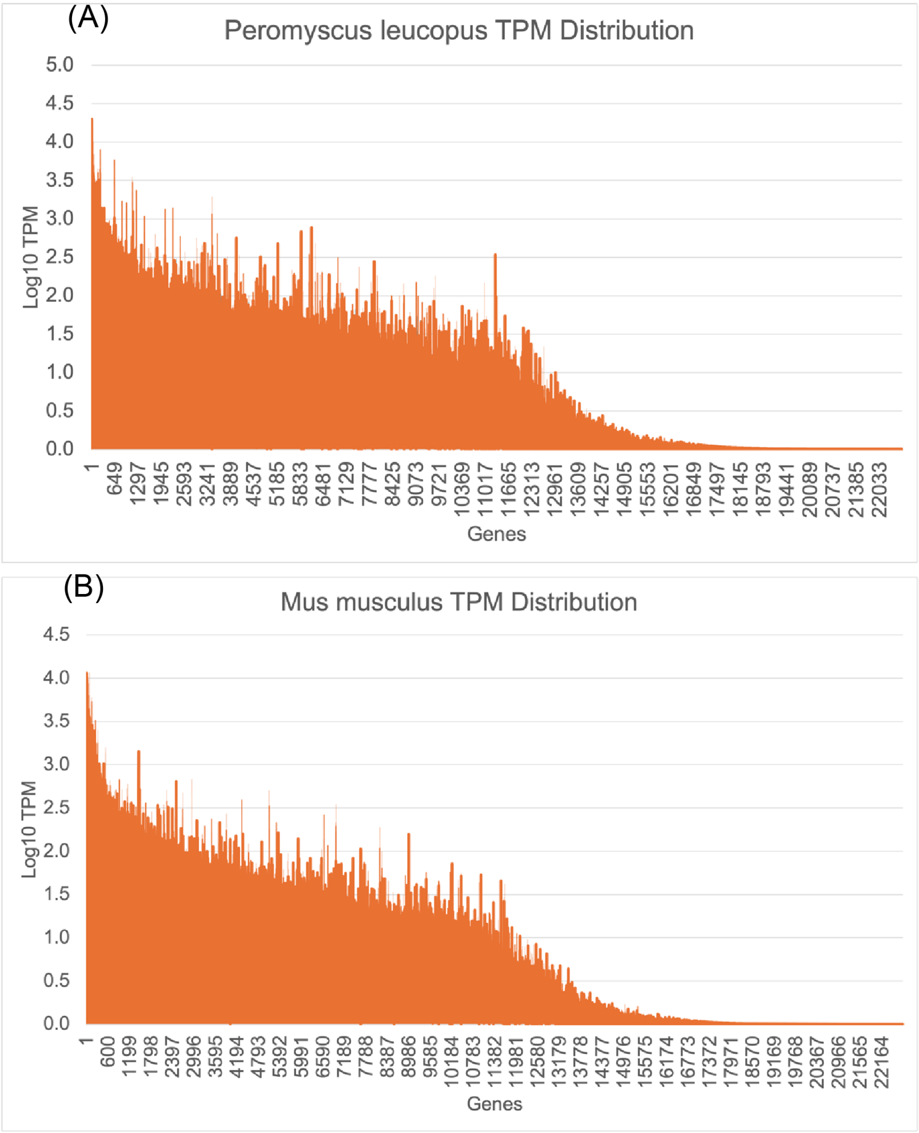

Figure S1. Histograms of the distributions of $\log_{10}$ of the mean (n=5) TPM values for 22,760 CDS of *Mus musculus* (panel A) and 22,654 CDS of *Peromyscus leucopus* (panel B) from genome-wide RNA-seq of primary dermal fibroblast cultures under control conditions. The *x*-axis is the cumulative CDS count. Data for analysis are in Dryad Tables D1 and D2.
