# Supplementary material for: Dermal fibroblast cultures recapitulate differences between deermice and mice in their responses to a Toll-like receptor agonist
Source: Front Immunol. 2025 Nov 4;16:1666789. doi: 10.3389/fimmu.2025.1666789 (PMC12623179; doi:10.3389/fimmu.2025.1666789)
Supplement: Supplementary file 6 [file Image3.pdf]

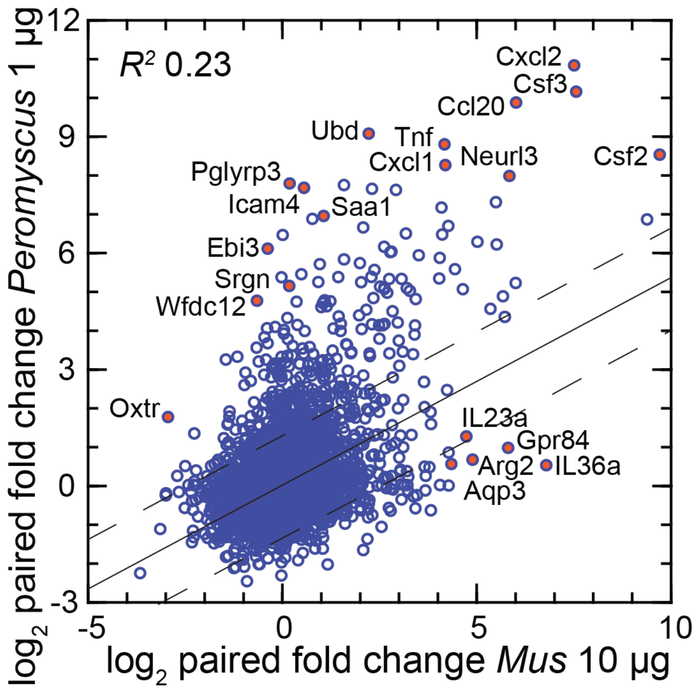

Figure S3

Figure S3. Scatter plot of log-transformed FC values for each species against each other as described for Figure 2. The difference is that the comparison for *P. leucopus* is with *M. musculus* fibroblasts exposed to the 10 μg/ml concentration of Pam3CSK4. The linear regression line with 95% confidence interval and coefficient of determination (R^2) are shown. Selective genes that are up-regulated and differentially expressed (DEGs) for each species are indicated by name and red fill. Data for analysis are in Dryad Table D3.
